# Scalable Exsolution‐Derived E‐Ni/m‐MgAlO_x_ Catalysts with Anti‐Sintering Stability for Methane Dry Reforming

**DOI:** 10.1002/smll.202508028

**Published:** 2025-09-15

**Authors:** Kyung Hee Oh, Shin Wook Kang, Byeong‐Seon An, Jung‐Il Yang, Ji Chan Park

**Affiliations:** ^1^ Clean Fuel Research Laboratory Korea Institute of Energy Research Daejeon 34129 Republic of Korea; ^2^ Analysis Center for Energy Research Korea Institute of Energy Research Daejeon 34129 Republic of Korea

**Keywords:** anti‐sintering, exsolution, methane dry reforming, nanocatalyst, pilot‐scale synthesis

## Abstract

Sintering and carbon deposition are primary causes of deactivation in Ni‐based catalysts during methane dry reforming (MDR). To address these issues, a scalable exsolution‐derived catalyst, E‐Ni/m‐MgAlO_x_, is reported, consisting of ≈9 nm Ni nanoparticles exsolved within a Mg‐doped mesoporous alumina matrix via a sol–gel method. This synthesis strategy ensures uniform precursor mixing and controlled exsolution, producing firmly anchored Ni nanoparticles with strong metal‐support interactions that effectively suppress sintering and coking. The incorporation of Mg promotes the formation of a stable MgAl_2_O_4_ spinel phase and enhances surface basicity, thereby facilitating efficient CO_2_ activation. The catalyst demonstrates high activity and excellent long‐term stability under high‐temperature MDR conditions (800 °C–900 °C, CH_4_/CO_2_/N_2_ = 1:1:8, >1000 h), achieving a cumulative greenhouse gas conversion exceeding 20000 L·g_cat_^−1^. Moreover, the synthesis process is straightforward, scalable, and highly promising for improving the durability of Ni‐based catalysts.

## Graphical abstract

A sintering‐resistant E‐Ni/m‐MgAlO_x_ catalyst with exsolved Ni nanoparticles shows >1000 h stability in methane dry reforming, achieving cumulative greenhouse gas conversion above 20000 L·g_cat_^−1^ at 800 °C–900 °C.

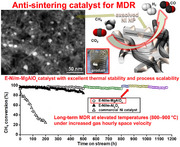

## 1 Introduction

Sintering of active metal nanoparticles remains one of the most critical and persistent challenges in high‐temperature catalytic reactions.^[^ ^1^, ^2^, ^3^ ^]^ Catalysts operating under elevated thermal conditions often suffer from nanoparticle aggregation, detachment from the support, and carbon deposition, which lead to rapid deactivation and reduced operational stability.^[^ ^4^, ^5^, ^6^ ^]^ These structural degradations hinder long‐term catalytic performance and remain a key limitation for both industrial and academic applications.^[^ ^7^, ^8^, ^9^ ^]^

As the global demand for efficient carbon‐neutral technologies continues to rise, there is growing interest in catalytic processes capable of converting greenhouse gases into value‐added products. Among them, methane dry reforming (MDR), which converts CH_4_ and CO_2_ into syngas (H_2_ and CO), stands out as it simultaneously reduces emissions and produces a valuable chemical feedstock.^[^ ^10^, ^11^, ^12^ ^]^ However, the commercial viability of MDR remains constrained by the poor thermal stability of conventional catalysts under industrial operating conditions.

Ni‐based catalysts have been extensively explored for MDR because of their high intrinsic activity and cost‐effectiveness.^[^ ^13^, ^14^, ^15^ ^]^ Despite these advantages, Ni catalysts tend to degrade at high temperatures above 800 °C, primarily due to irreversible sintering of metal nanoparticles and the formation of graphitic carbon deposits.^[^ ^16^, ^17^, ^18^ ^]^ These deactivation mechanisms lead to a significant decline in catalytic performance and can cause severe reactor issues, such as clogging. Therefore, the development of thermally robust Ni‐based catalysts that can endure prolonged exposure to harsh MDR conditions remains a key challenge in heterogeneous catalysis.^[^ ^19^, ^20^ ^]^

Strategies to improve the thermal stability and catalytic efficiency of Ni‐based systems include controlling the crystal size of active Ni, tailoring the structure and properties of support materials, and modifying surface chemistry through alloy design.^[^ ^21^, ^22^, ^23^, ^24^, ^25^ ^]^ Recent efforts have also focused on enhancing metal‐support interactions and constructing advanced nanostructures through innovative synthesis methods. Among the various stabilization strategies, exsolution has recently gained considerable attention as an effective approach for anchoring catalytic nanoparticles. Under reducing conditions, metal species emerge in situ from an oxide lattice, forming nanoparticles that are partially socketed into the support. These embedded interfaces provide strong metal‐support interactions, offering remarkable resistance to sintering, migration, and detachment even under high‐temperature stress.^[^ ^26^, ^27^, ^28^, ^29^, ^30^ ^]^

Building on these principles, exsolution‐based catalysts have been widely explored in perovskite‐ and dense oxide‐derived systems, where exsolved nanoparticles exhibit strong metal‐support interactions and provide remarkable resistance to sintering and coking under harsh MDR conditions.^[^ ^31^, ^32^ ^]^ Nevertheless, these dense oxide systems inherently possess low surface areas and suffer from mass‐transport limitations, which restrict their catalytic activity and pose significant challenges for scale‐up and industrial implementation.

In this work, we introduce a scalable exsolution‐derived catalyst, E‐Ni/m‐MgAlO_x_, in which ≈9 nm Ni nanoparticles are exsolved within a Mg‐doped mesoporous alumina matrix prepared via a sol–gel method. The mesoporous alumina framework provides high surface area, efficient gas diffusion, and uniform Ni dispersion, while Mg incorporation promotes the formation of a stable MgAl_2_O_4_ spinel phase and enhances surface basicity, thereby facilitating CO_2_ activation. Importantly, the sol–gel synthesis is readily scalable, as demonstrated by the successful production of hundreds of grams of the catalyst with preserved structure and performance. To the best of our knowledge, this study represents the first demonstration of a scalable exsolution strategy applied to mesoporous catalyst systems with tunable compositions, achieving near‐equilibrium conversions and exceptional long‐term stability (> 1000 h) under industrially relevant MDR conditions.

## 2 Results and Discussion

**Figure 1:**
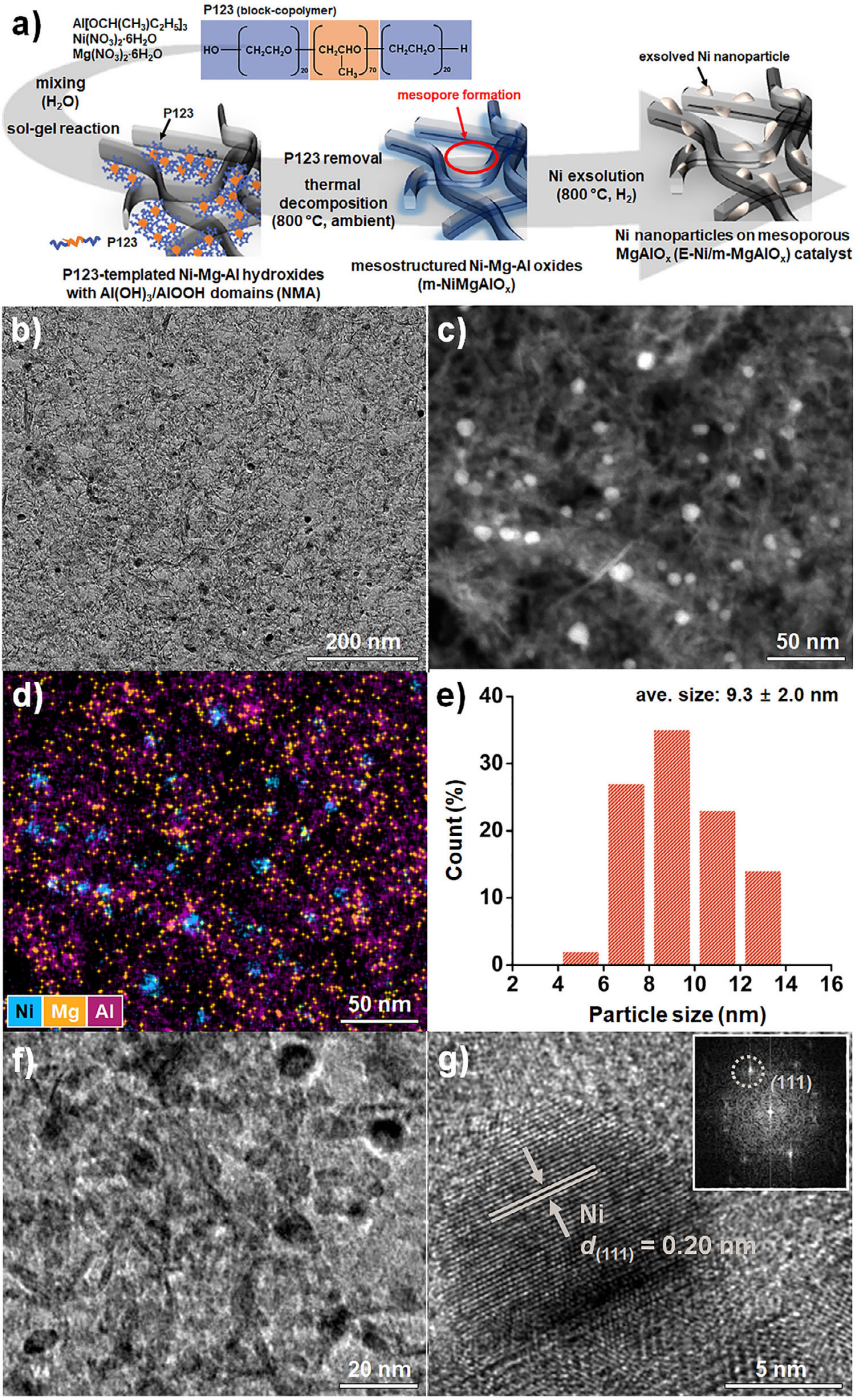
a) Schematic illustration of the synthesis process for the E‐Ni/m‐MgAlO_x_ catalyst, b) TEM image of its mesoporous structure, c) HAADF‐TEM image, d) Elemental mapping images of Ni, Mg, and Al, e) Particle size distribution of Ni nanoparticles, f) High‐magnification TEM image showing embedded Ni particles, and g) HR‐TEM image of a single Ni nanoparticle with the corresponding FFT pattern (inset). Images b–g) show the structural and morphological characteristics of the E‐Ni/m‐MgAlO_x_ catalyst.

The E‐Ni/m‐MgAlO_x_ nanocatalyst was synthesized via a sol–gel method using Pluronic P123 as a soft template, followed by calcination and hydrogen‐induced exsolution (**Figure** **1**a). The precursor sol was prepared by dissolving nickel nitrate hexahydrate, magnesium nitrate hexahydrate, and aluminum tri‐sec‐butoxide in an acidic aqueous solution containing Pluronic P123. Acid‐catalyzed hydrolysis of aluminum tri‐sec‐butoxide produced Al‐OH species, while Ni(II) and Mg(II) ions were homogeneously distributed through complete dissociation of their respective salts. Subsequent condensation reactions led to the formation of a crosslinked inorganic framework uniformly incorporated into the Pluronic P123 micelle network via chelation with metal ions.^[^ ^33^, ^34^ ^]^ This process yielded a Ni‐Mg‐Al hydroxide gel containing Al(OH)_3_/AlOOH domains (NMA), as confirmed by transmission electron microscopy (TEM) and X‐ray diffraction (XRD) (Figure S1, Supporting Information). Calcination at 800 °C in ambient air decomposed the P123 template, resulting in the formation of well‐defined mesopores and the production of mesostructured Ni‐Mg‐Al mixed oxides (m‐NiMgAlO_x_).

TEM and elemental mapping of m‐NiMgAlO_x_ revealed a well‐ordered mesoporous architecture with uniformly distributed Ni, Mg, and Al elements (Figure S2, Supporting Information). Upon hydrogen reduction, metallic Ni nanoparticles were exsolved from the oxide matrix, yielding the final E‐Ni/m‐MgAlO_x_ catalyst. TEM images confirmed the uniform distribution of Ni particles throughout the mesoporous framework (Figure 1b). High‐angle annular dark‐field (HAADF)‐TEM and elemental mapping further verified the dense and homogeneous dispersion of Ni nanoparticles without observable aggregation (Figure 1c,d). Based on the measurements of 200 particles, the average Ni particle diameter was determined to be 9.3  ±  2.0 nm, exhibiting a narrow size distribution without the use of external stabilizers (Figure 1e). A higher‐magnification TEM image revealed that the Ni particles were well‐defined, spherical, and embedded within the oxide matrix (Figure 1f). A high‐resolution TEM (HR‐TEM) image of an individual Ni nanoparticle revealed clear lattice fringes with an interplanar spacing of 0.20 nm, corresponding to the (111) planes of face‐centered cubic (fcc) Ni, and the corresponding fast Fourier transform (FFT) pattern confirmed its crystalline structure (Figure 1g).

**Figure 2:**
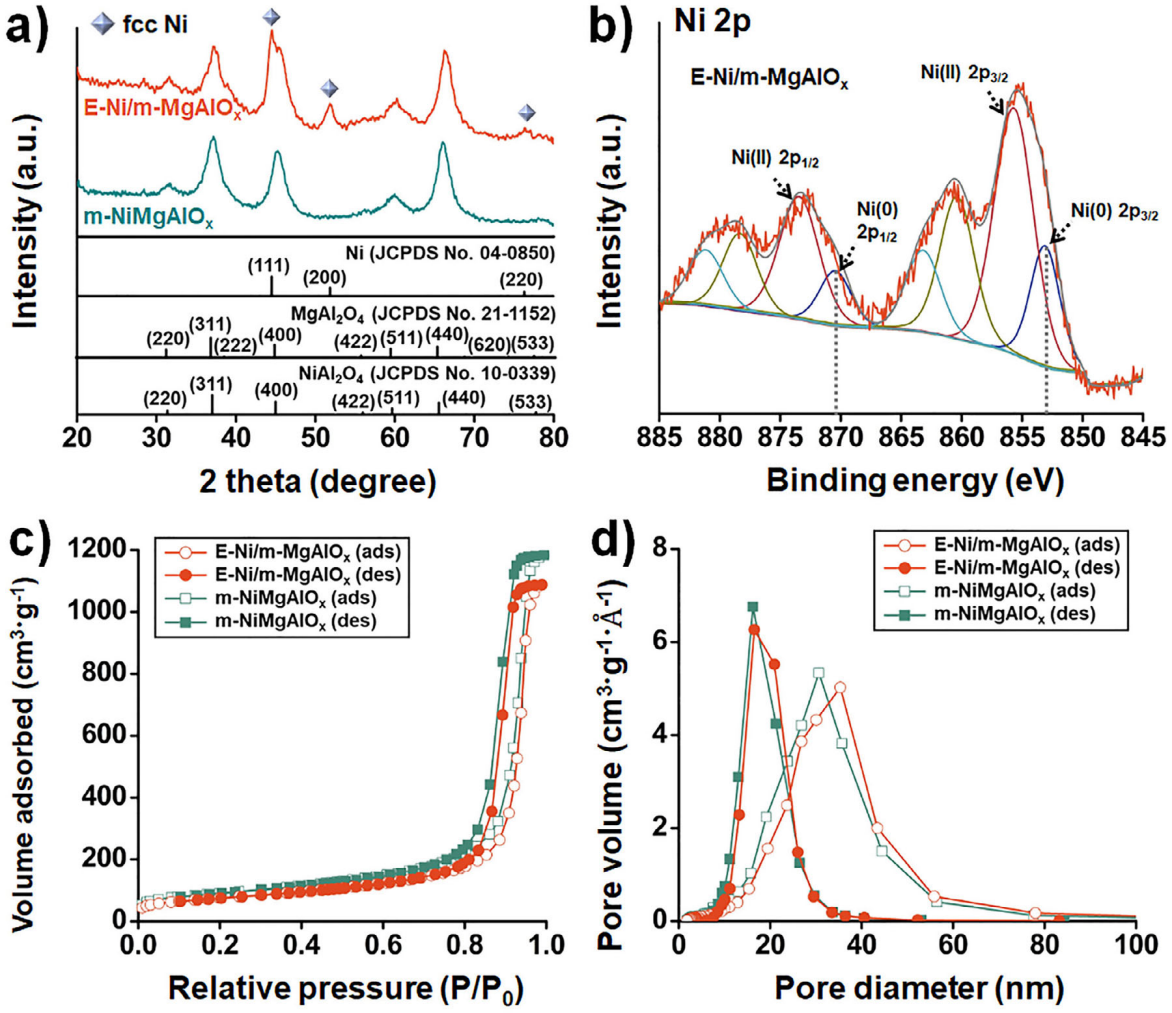
a) XRD patterns of m‐NiMgAlO_x_ and E‐Ni/m‐MgAlO_x_, b) XPS spectrum of E‐Ni/m‐MgAlO_x_ in the Ni 2p region, c) N_2_ adsorption–desorption isotherms of m‐NiMgAlO_x_ and E‐Ni/m‐MgAlO_x_, and d) Pore size distribution curves derived from the adsorption and desorption branches using the BJH method. The dotted lines in b) indicate the binding energy positions of metallic Ni, based on reference data from the Handbook of X‐ray Photoelectron Spectroscopy.

XRD analysis of m‐NiMgAlO_x_ exhibited diffraction peaks corresponding to spinel‐type MgAl_2_O_4_ and NiAl_2_O_4_ phases (JCPDS Nos. 21–1152 and 10–0339), confirming the successful formation of a mixed oxide framework (**Figure** **2**a). After hydrogen reduction, additional peaks appeared in the E‐Ni/m‐MgAlO_x_ sample that matched the fcc structure of metallic Ni (JCPDS No. 04–0850), confirming the formation of single‐crystalline Ni nanoparticles via exsolution. The crystallite size of Ni, estimated using the Debye–Scherrer equation based on the (200) reflection, was approximately 7.8 nm. Furthermore, HR‐TEM and FFT analyses revealed lattice fringes indexed to the (220) and (022) planes with a [101] zone axis, providing direct microscopic evidence of MgAl_2_O_4_ spinel formation (Figure S3, Supporting Information).

Further information on the Ni oxidation states was obtained via X‐ray photoelectron spectroscopy (XPS) (Figure 2b). The reduced final E‐Ni/m‐MgAlO_x_ sample exhibited deconvoluted peaks at 853.0 eV (Ni 2p_3/2_) and 870.3 eV (Ni 2p_1/2_), corresponding to Ni(0), along with residual signals from Ni(II) and associated satellite features. These results confirmed the successful exsolution of catalytically active metallic Ni. Although ethanol was used to passivate the fresh E‐Ni/m‐MgAlO_x_ catalyst powders, partial oxidation of the highly reactive Ni nanoparticles occurred during sample handling due to exposure to air. In contrast, the unreduced m‐NiMgAlO_x_ sample displayed only Ni(II) peaks at 855.7 and 873.2 eV, accompanied by intense satellite features (Figure S4, Supporting Information), confirming that nickel was fully oxidized prior to reduction. In NiAl_2_O_4_, Ni(II) showed a higher binding energy in XPS than in NiO due to its different chemical environment. Surrounded by electronegative Al(III) ions in the spinel structure, Ni loses electron density, leading to stronger core electron binding and thus higher binding energy.^[^ ^35^, ^36^ ^]^ The observed shift in binding energy from Ni(II) to Ni(0) reflects changes in the electronic environment and the emergence of strong metal‐support interactions during the exsolution process.

Textural properties were evaluated using nitrogen sorption isotherms and Barrett‐Joyner‐Halenda (BJH) analysis. Both m‐NiMgAlO_x_ and E‐Ni/m‐MgAlO_x_ samples exhibited high Brunauer‐Emmett‐Teller (BET) surface areas of 326.2 and 268.9 m^2^∙g^−1^, respectively, along with large total pore volumes of 1.8 and 1.7 cm^3^∙g^−1^ (Figure 2c). BJH analysis showed average pore diameters of 30.7 nm (adsorption branch) and 16.2 nm (desorption branch) for the oxide, and 35.3 and 16.5 nm for the catalyst, respectively (Figure 2d), confirming the presence of well‐developed mesoporosity favorable for enhanced mass transport and catalytic performance. Inductively Coupled Plasma Optical Emission Spectrometry (ICP‐OES) analysis confirmed that the final Ni content in the E‐Ni/m‐MgAlO_x_ catalyst was 4.3 wt.%, while the Mg content was 2.7 wt.%. These values closely match the theoretical compositions of 4.54 wt.% for Ni and 2.74 wt.% for Mg, based on the precursor formulation. Overall, the results demonstrate that the sol–gel‐derived E‐Ni/m‐MgAlO_x_ catalyst retained its mesoporous structure following high‐temperature treatment and successfully incorporated well‐dispersed, catalytically active Ni nanoparticles. The robust MgAlO_x_ matrix enhanced metal‐support interactions, effectively suppressing particle sintering and aggregation.

**Figure 3:**
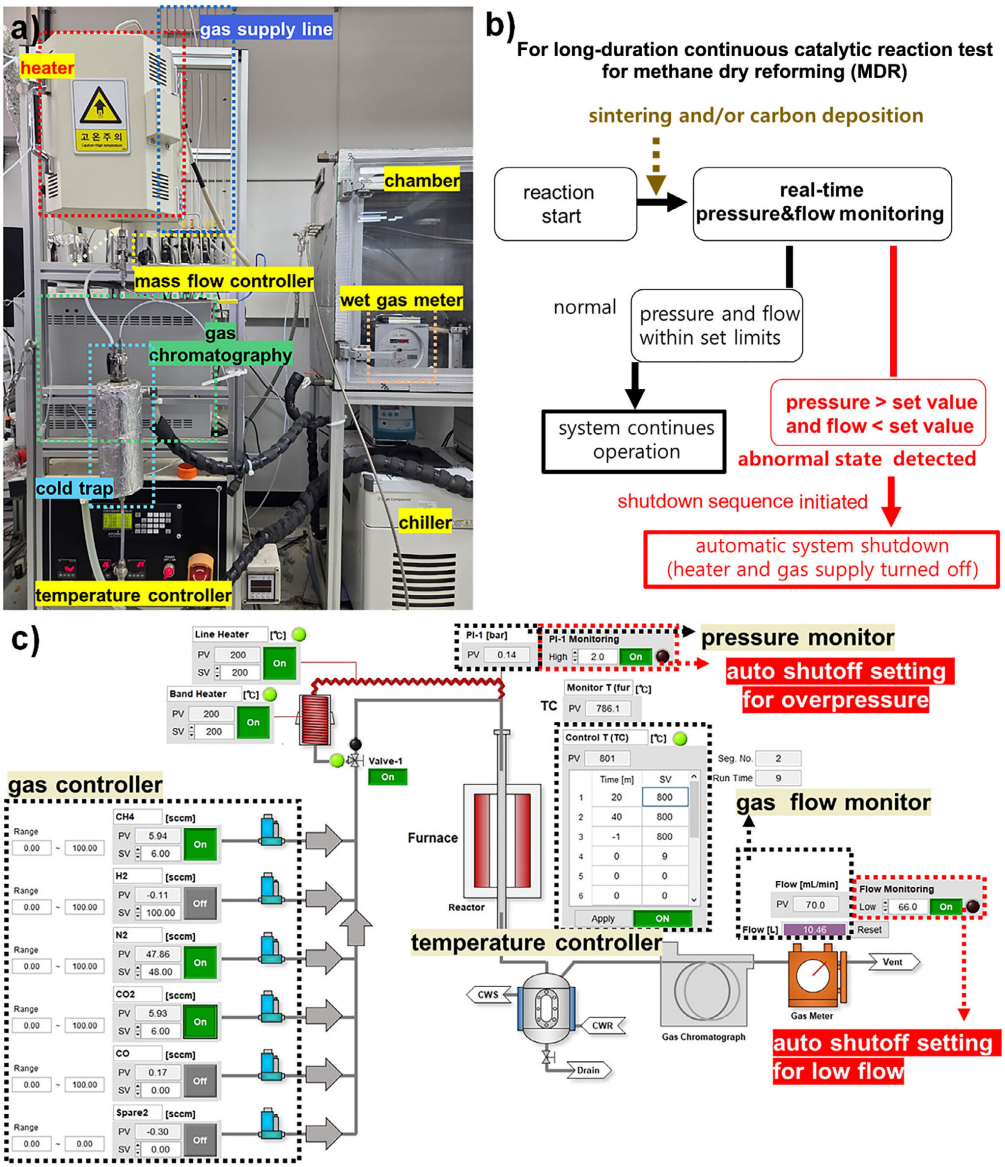
a) Photograph of the continuous MDR reaction system, b) schematic diagram of the automatic shutdown mechanism activated by abnormal pressure or gas flow, and c) control interface displaying safety functions for overpressure, low gas flow, and communication errors.

To assess the performance and stability of E‐Ni/m‐MgAlO_x_ catalyst under practical conditions, a custom‐built reaction system was developed for safe and continuous long‐term MDR testing (**Figure** **3**a). High‐temperature MDR often leads to catalyst deactivation or reactor blockage, presenting significant challenges in both research and industrial applications. To mitigate these issues, an automated platform with programmable shutdown logic was implemented. The system enables real‐time monitoring of key parameters such as gas flow rate and reactor pressure, and automatically responds to deviations predefined beyond safety thresholds (Figure 3b). If product gas flow drops or internal pressure rises abnormally due to catalyst degradation or carbon accumulation, the system triggers an emergency shutdown to stop both heating and gas supply. The control logic, executed via a programmable automation system, incorporates multiple fail‐safes: overpressure and low‐flow protection, as well as interface error detection to protect against sensor or communication failures (Figure 3c).

**Figure 4:**
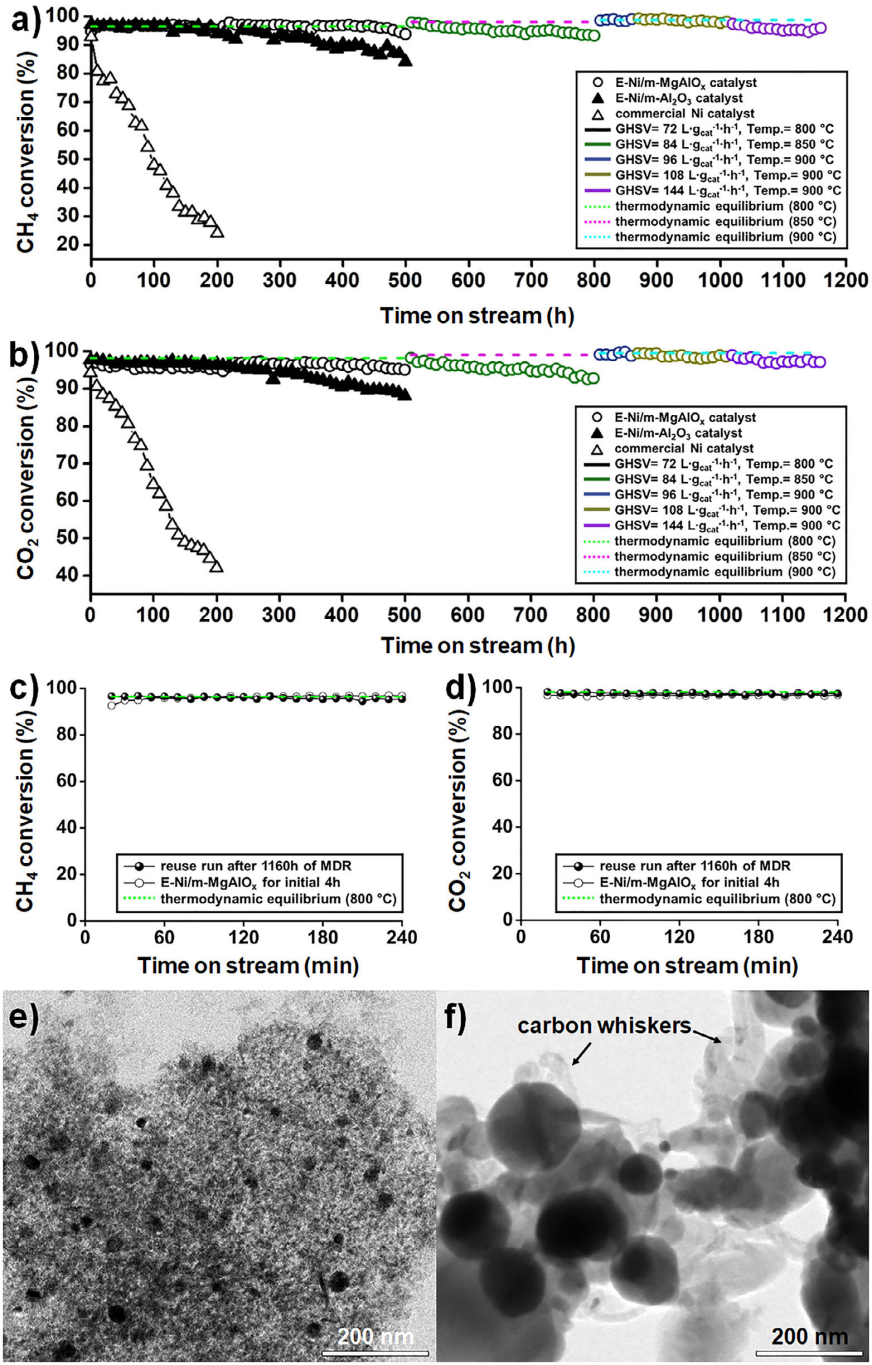
a,b) CH_4_ and CO_2_ conversions over time for each catalyst under long‐term MDR conditions (CH_4_/CO_2_/N_2_ = 1/1/8, GHSV = 72–144 L∙g_cat_^−1^∙h^−1^, at 800, 850, and 900 °C). c,d) CH_4_ and CO_2_ conversions during the reuse test of the E‐Ni/m‐MgAlO_x_ catalyst under the initial reaction conditions (GHSV = 72 L∙g_cat_^−1^∙h^−1^) following 1160 h of MDR operation. e) TEM image of the E‐Ni/m‐MgAlO_x_ catalyst recovered after 1164 h of reaction. f) TEM image of the commercial Ni catalyst recovered after 200 h of reaction. Thermodynamic equilibrium values at 800–900 °C (dashed lines in a–d) were calculated using HSC Chemistry 9.0.

The performance of the E‐Ni/m‐MgAlO_x_ catalyst was evaluated in a continuous MDR test conducted over 1160 h. To assess its stability under increasingly harsh conditions, the gas hourly space velocity (GHSV) was gradually increased from 72 to 144 L∙g_cat_^−1^∙h^−1^, while the reaction temperature was raised from 800 to 900 °C. The test began at 800 °C and a GHSV of 72 L∙g_cat_^−1^∙h^−1^, using a CH_4_/CO_2_ ratio of 1 with N_2_ as the diluent gas. During the initial 500 h, the catalyst exhibited excellent stability, maintaining CH_4_ and CO_2_ conversions of 93.7% and 95.0% at time‐on‐stream (TOS) = 500 h, respectively, which are close to the thermodynamic equilibrium values (CH_4_: 96.5%, CO_2_: 98.2%). Further testing under increasingly severe conditions confirmed the catalyst's robustness. At 850 °C and a GHSV of 84 L∙g_cat_^−1^∙h^−1^, high conversion levels were sustained, with CH_4_ and CO_2_ conversions of 93.4% and 92.6% at TOS = 800 h, respectively. At 900 °C and a GHSV of 96 L∙g_cat_^−1^∙h^−1^, initial conversions reached 98.5% (CH_4_) and 99.2% (CO_2_), remaining stable at 98.9% and 98.8% after 60 h. Even at a GHSV of 108 L∙g_cat_^−1^∙h^−1^, the catalyst maintained conversions near equilibrium throughout a 150 h run. At the highest tested GHSV of 144 L∙g_cat_^−1^∙h^−1^ at 900 °C, CH_4_ and CO_2_ conversions of 95.9% and 97.2% were achieved at TOS = 1160 h. Overall, the E‐Ni/m‐MgAlO_x_ catalyst demonstrated excellent long‐term stability and cumulative greenhouse gas conversion performance, with CO_2_ conversion consistently maintained at 97%–99% of the thermodynamic equilibrium values throughout the entire 1160 h operation (**Figure** **4**a,b; Table S1, Supporting Information).

The E‐Ni/m‐MgAlO_x_ catalyst achieved a total reactant conversion of 20316 L∙g_cat_^−1^, demonstrating excellent productivity under long‐term reaction conditions. This value surpasses that of most previously reported catalysts, and is particularly notable because it was achieved under moderate GHSV conditions (72–144 L∙g_cat_^−1^∙h^−1^) while maintaining near‐equilibrium conversions, indicating strong practical potential. Although NiES (Ni embedded in SiO_2_) exhibited the highest turnover number for CO_2_, this result was obtained under extremely high GHSV conditions (250–600 L∙g_cat_^−1^∙h^−1^), thereby limiting its applicability to direct industrial comparisons.^[^ ^37^ ^]^ Other catalysts, such as LCFN55 and Ni/SBA‐15/FeCrAl, achieved conversions of approximately 28125 and 24864 L∙g_cat_^−1^, respectively, but relied on complex support structures that may hinder scalability.^[^ ^29^, ^38^ ^]^

For comparison, a Mg‐free counterpart was synthesized using the same procedure, except for the omission of the Mg precursor in the initial step. Thermal reduction of mesostructured Ni–Al oxides (m‐NiAlO_x_) yielded the final catalyst, consisting of exsolved Ni nanoparticles supported on mesoporous alumina (E‐Ni/m‐Al_2_O_3_). TEM analysis revealed a uniform dispersion of Ni nanoparticles with an average size of 8.1 ± 1.8 nm (Figure S5a–d, Supporting Information). In the XRD patterns of m‐NiAlO_x_, major diffraction peaks were observed at 2*θ* = 37.0°, 45.0°, and 65.5°, corresponding to the (311), (400), and (440) planes of face‐centered cubic NiAl_2_O_4_ (JCPDS No. 10–0339) (Figure S5e, Supporting Information). After reduction, E‐Ni/m‐Al_2_O_3_ exhibited additional peaks at 44.5° and 51.8°, which were assigned to the (111) and (200) planes of metallic Ni. Based on the (200) reflection, the Ni crystallite size was estimated to be 7.6 nm using the Debye‐Scherrer equation. N_2_ sorption measurements at −196 °C showed a type IV adsorption–desorption hysteresis loop for E‐Ni/m‐Al_2_O_3_ (Figure S5f, Supporting Information). The BET surface area was determined to be 265 m^2^∙g^−1^, and the total pore volume was 1.7 cm^3^∙g^−1^, both values comparable to those of E‐Ni/m‐MgAlO_x_.

To investigate the surface acid–base properties of the catalysts, ammonia temperature‐programmed desorption (NH_3_‐TPD) and carbon dioxide temperature‐programmed desorption (CO_2_‐TPD) analyses were performed. The NH_3_‐TPD profiles exhibited three major desorption peaks, corresponding to weak acid sites at 150–250 °C, medium‐strength acid sites at 250–400 °C, and strong acid sites at 400–700 °C (Figure S6a,b, Supporting Information). The E‐Ni/m‐Al_2_O_3_ catalyst displayed a higher density of medium‐strength acid sites compared with the E‐Ni/m‐MgAlO_x_ catalyst. In contrast, the CO_2_‐TPD spectra showed distinct desorption peaks at 50–150 °C and 200–400 °C, which can be attributed to weak and medium basic sites, respectively (Figure S6c, Supporting Information). Notably, the E‐Ni/m‐MgAlO_x_ catalyst possessed a greater number of basic sites than the undoped E‐Ni/m‐Al_2_O_3_ catalyst (Figure S6d, Supporting Information). These results indicate that Mg incorporation effectively suppresses medium‐strength acidity while enhancing basicity.

**Figure 5:**
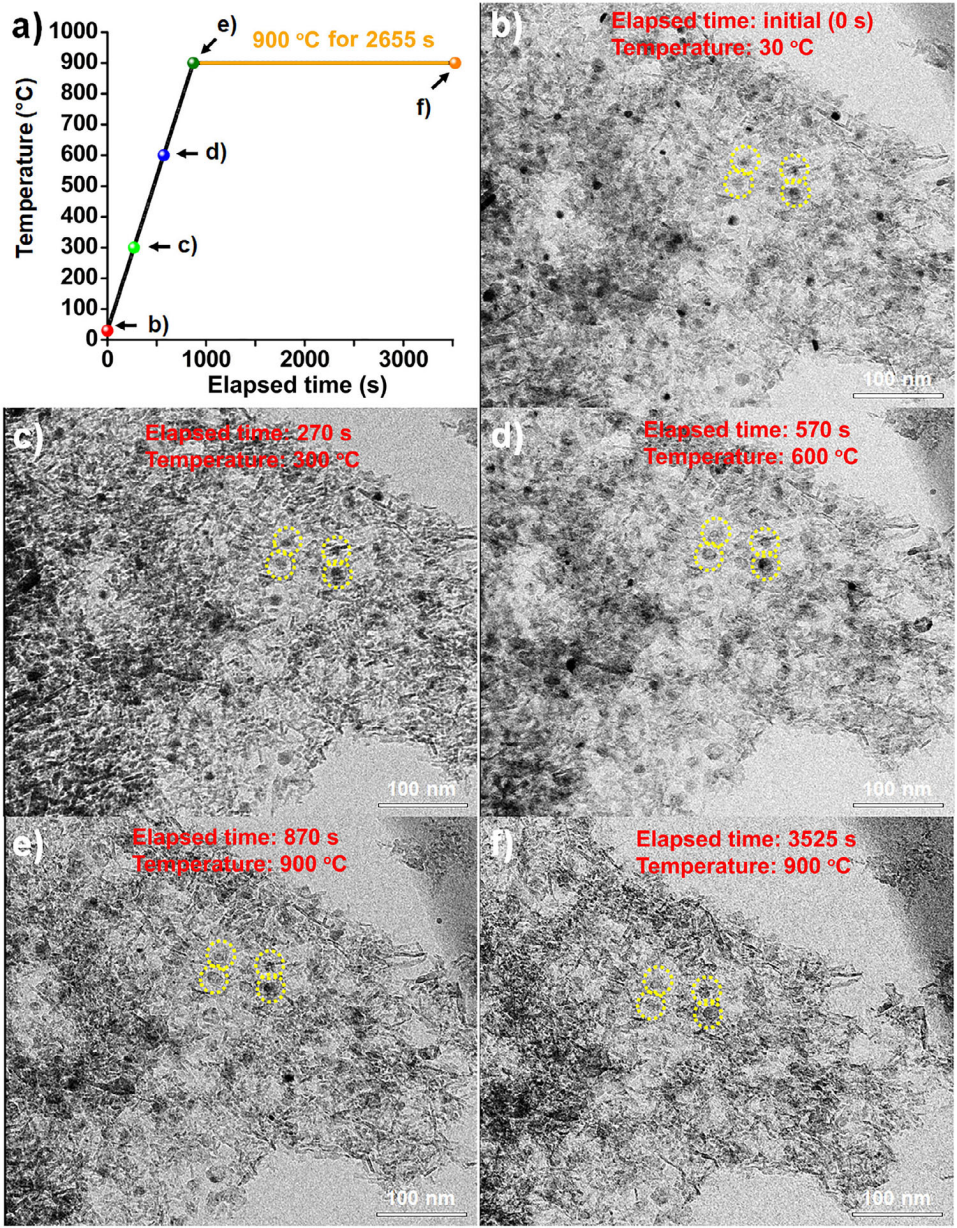
a) Temperature profile during the in situ TEM heating experiment. b–f) In situ TEM images of the E‐Ni/m‐Al_2_O_3_ catalyst acquired at different temperatures and elapsed times. Yellow dashed circles in the TEM images indicate the locations of representative Ni particles.

The high‐temperature sintering stability of the E‐Ni/m‐Al_2_O_3_ catalyst was further evaluated by in situ TEM experiments, in which particle behavior was monitored under the same conditions applied to the commercial Ni catalyst. The sample was heated from room temperature to 900 °C and then maintained at this temperature for 2655 s, resulting in a total elapsed time of 3525 s (**Figure** **5**a). The corresponding TEM images (Figure 5b–f) illustrate the evolution of Ni nanoparticles during this process. At the initial state (30 °C, Figure 5b), the Ni nanoparticles were uniformly dispersed with small sizes. Upon heating to 300 °C (270 s, Figure 5c) and 600 °C (570 s, Figure 5d), the particles remained stable without noticeable growth or aggregation. Even after reaching 900 °C (870 s, Figure 5e), the Ni nanoparticles largely retained their morphology with only minor size changes. Remarkably, after being held at 900 °C for 2655 s (total elapsed time 3525 s, Figure 5f), the particles still exhibited minimal growth, confirming their strong resistance to sintering (Movie S1, Supporting Information). This stability is in stark contrast to the significant particle growth and agglomeration observed for the commercial Ni catalyst under identical conditions (Figure S7, Movie S2, Supporting Information).

Despite the excellent thermal stability observed by in situ TEM, the E‐Ni/m‐Al_2_O_3_ catalyst exhibited a gradual decline in performance after 200 h of continuous MDR operation (Figure 4a,b). This deactivation is likely due to the limited structural stability of the alumina support under prolonged high‐temperature conditions in the absence of magnesia, as well as progressive carbon deposition facilitated by the acidic nature of the alumina surface. In contrast, the commercial Ni catalyst exhibited a much more rapid decline in performance under the same reaction.

Conditions (800 °C, GHSV = 72 L∙g_cat_^−1^∙h^−1^), with CH_4_ and CO_2_ conversions dropping to 24.1% and 42.0% at TOS = 200 h, respectively. This pronounced deactivation is likely caused by the simultaneous occurrence of nanoparticle sintering and carbon deposition, which progressed more severely in the absence of structural and chemical stabilization.

To further assess the extent of deactivation of the E‐Ni/m‐MgAlO_x_ catalyst after long‐term operation, the spent catalyst was directly reused under the initial reaction conditions (800 °C, GHSV = 72 L∙g_cat_^−1^∙h^−1^), for an additional 4 h following the 1160 h reaction. The CH_4_ and CO_2_ conversions measured during this reuse test were nearly identical to those observed in the initial 4 h run, confirming that the catalyst retained its original activity at levels close to thermodynamic equilibrium (Figure 4c,d). These results clearly demonstrate that the E‐Ni/m‐MgAlO_x_ catalyst preserved its intrinsic performance even after prolonged exposure to harsh MDR conditions.

To verify structural stability, TEM analysis was conducted on the E‐Ni/m‐MgAlO_x_ catalyst recovered after the long‐term reaction and subsequent 4 h reuse test. Even after 1164 h of operation, low‐magnification TEM images revealed uniformly dispersed Ni nanoparticles with an average size of ≈20 nm (Figure 4e). In contrast, the commercial Ni catalyst recovered after only 200 h exhibited severely sintered Ni particles several hundred nanometers in size, accompanied by carbon whiskers formed from extensive carbon deposition (Figure 4f). These results suggest that the exsolved Ni nanoparticles in E‐Ni/m‐MgAlO_x_ are strongly anchored through robust metal‐support interactions, while MgO‐derived basicity plays a crucial role in suppressing carbon accumulation. In addition, the fresh E‐Ni/m‐MgAlO_x_ sample displayed a rod‐like porous alumina structure with well‐dispersed Ni nanoparticles, while the post‐reaction HR‐TEM images confirmed the preservation of the MgAl_2_O_4_ spinel phase, with distinct lattice fringes and FFT patterns (Figure S8, Supporting Information). This demonstrates that the MgAl_2_O_4_ framework remained structurally intact during long‐term MDR operation.

For comparison, TEM analyses of the undoped E‐Ni/m‐AlO_x_ catalyst before and after 500 h of MDR operation revealed pronounced structural degradation. While the fresh catalyst displayed uniformly dispersed Ni nanoparticles (Figure S9a,b, Supporting Information), the spent sample exhibited partial aggregation and graphitic carbon shells surrounding the Ni particles (Figure S9c,d, Supporting Information). Raman spectra further confirmed graphitic carbon deposition, with distinct D (≈1350 cm^−1^) and G (≈1590 cm^−1^) bands and an *I*_G_/*I*_D_ ratio of 0.75 (Figure S9e, Supporting Information). These results indicate that the deactivation of the undoped catalyst is likely caused by excessive acidity and insufficient basicity, which accelerate coke formation and limit CO_2_ activation, ultimately leading to support degradation, nanoparticle sintering, and graphitic carbon growth under prolonged high‐temperature conditions. By contrast, Ni nanoparticles in the Mg‐doped E‐Ni/m‐MgAlO_x_ catalyst remained well‐anchored within the support with only limited particle growth, underscoring the stabilizing effect of Mg incorporation in preserving structural integrity during long‐term MDR operation.

**Figure 6:**
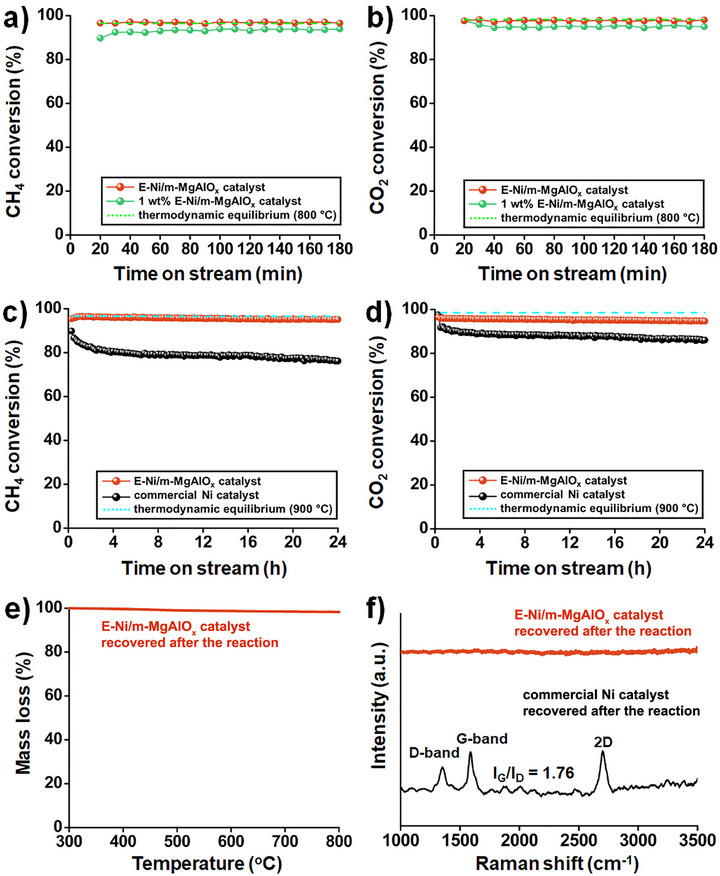
a) CH_4_ conversion of E‐Ni/m‐MgAlO_x_ and a low‐Ni‐loading (1 wt.%) catalyst at 800 °C (CH_4_/CO_2_/N_2_ = 1:1:8, GHSV = 72 L·g_cat_^−1^·h^−1^). b) CO_2_ conversion under the same conditions as in (a). c) CH_4_ conversion of E‐Ni/m‐MgAlO_x_ and a commercial Ni catalyst at 900 °C for 24 h under undiluted conditions (CH_4_/CO_2_ = 1:1, GHSV = 54 L·g_cat_^−1^·h^−1^). d) CO_2_ conversion under the same conditions as in (c). e) TGA profile of the recovered E‐Ni/m‐MgAlO_x_ catalyst after the reaction (900 °C, 24 h, CH_4_/CO_2_ = 1:1, GHSV = 54 L·g_cat_^−1^·h^−1^). f) Raman spectra of E‐Ni/m‐MgAlO_x_ and a commercial Ni catalyst recovered after the reaction (900 °C, 24 h, CH_4_/CO_2_ = 1:1, GHSV = 54 L·g_cat_^−1^·h^−1^).

Reducing the overall Ni loading in the catalyst can be an effective strategy to decrease particle size and enhance thermal stability. Based on this consideration, a series of experiments was conducted by varying the Ni loading. Catalysts with Ni loadings above 5 wt.% exhibited conversion performances nearly identical to that of the 5 wt.% sample, indicating a saturation in catalytic activity. In contrast, a slight reduction in CH_4_ and CO_2_ conversions was observed when the Ni content was decreased to 1 wt.% (**Figure** **6**a,b).

Further evaluation under undiluted reaction conditions at 900 °C with a GHSV of 54 L·g_cat_^−1^·h^−1^ confirmed that the 5 wt.% E‐Ni/m‐MgAlO_x_ catalyst maintained superior CH_4_ and CO_2_ conversion and stability compared to the commercial Ni catalyst (Figure 6c,d). In contrast, the 1 wt.% E‐Ni/m‐MgAlO_x_ catalyst showed a marked decrease in performance, primarily due to the low metal loading, which restricted both the number of active sites and the overall catalytic efficiency (Figure S10, Supporting Information).

To evaluate the extent of carbon deposition after the reaction, thermogravimetric analysis (TGA) and Raman spectroscopy were performed on the E‐Ni/m‐MgAlO_x_ catalyst recovered after 24 h of MDR at 900 °C under undiluted conditions (CH_4_/CO_2_ = 1:1, GHSV = 54 L·g_cat_^−1^·h^−1^). In the TGA profile, the E‐Ni/m‐MgAlO_x_ catalyst exhibited negligible mass loss, indicating that no significant carbon deposition occurred during the reaction (Figure 6e). Consistently, the absence of detectable Raman features for the E‐Ni/m‐MgAlO_x_ catalyst further confirms its strong resistance to carbon deposition under harsh MDR conditions (Figure 6f). By contrast, the Raman spectrum of the commercial Ni catalyst displayed pronounced D‐ and G‐bands with an I_G_/I_D_ ratio of 1.76, suggesting the accumulation of graphitic carbon.

**Figure 7:**
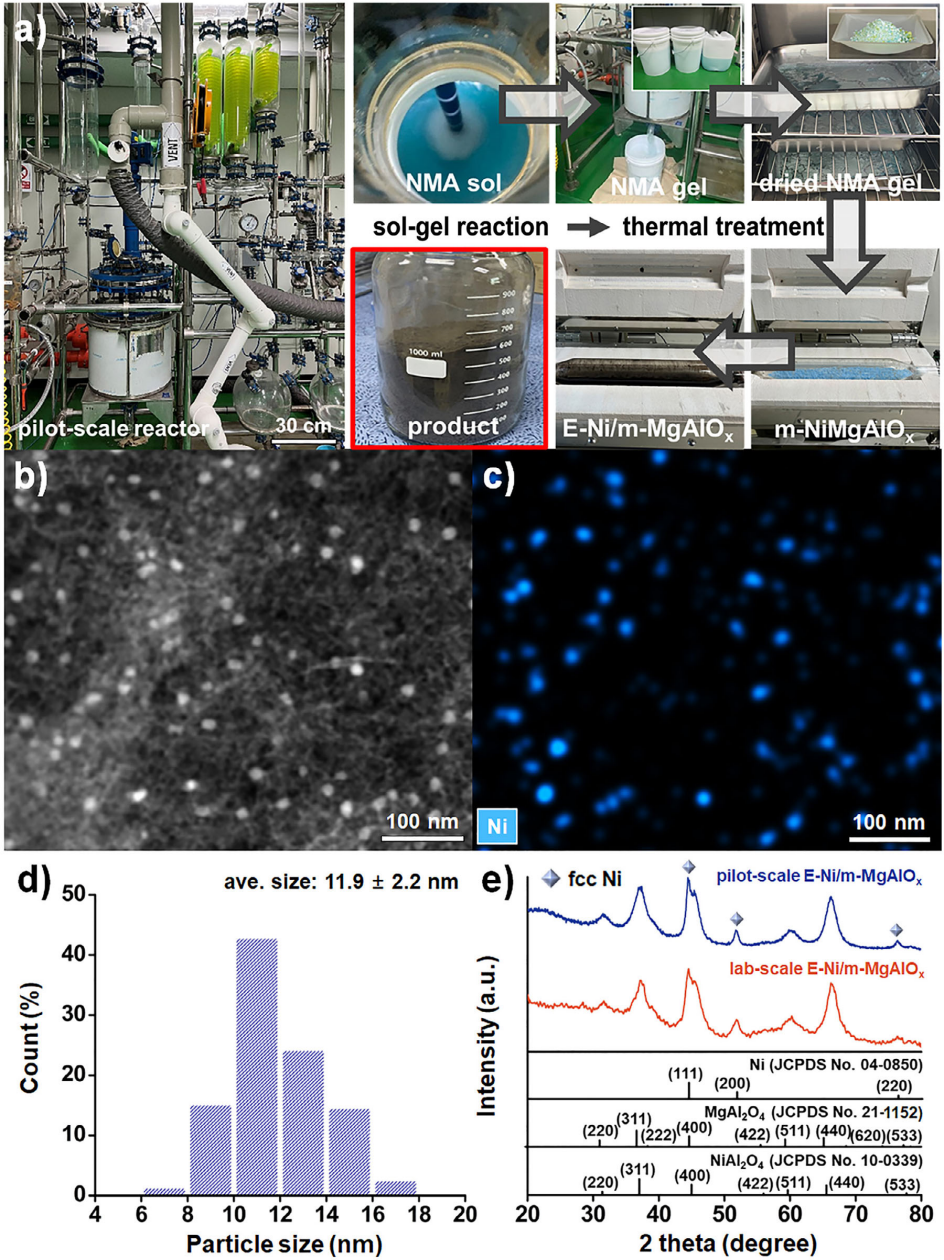
a) Photograph of the pilot‐scale synthesis process of the E‐Ni/m‐MgAlO_x_ catalyst. b) HAADF‐TEM image, c) elemental mapping of Ni, d) particle size distribution of Ni nanoparticles, and e) XRD patterns of lab‐ and pilot‐scale E‐Ni/m‐MgAlO_x_.

For industrial application of the MDR reaction, a robust catalyst that can be reproducibly synthesized and reliably produced on a large scale is essential. To achieve this, the synthesis of E‐Ni/m‐MgAlO_x_ was scaled up by 75 times from the laboratory scale to the kilogram level using a pilot‐scale process. Specifically, a large‐scale 60 L hydrothermal reactor was employed for the sol–gel process, followed by uniform high‐temperature calcination in a rotary tube furnace (**Figure** **7**a; Figure S11, Supporting Information). As in the lab‐scale process, the pilot‐scale synthesis proceeded through the formation of NMA via a sol–gel reaction, followed by template removal to yield m‐NiMgAlO_x_, and subsequent high‐temperature reduction under a H_2_ atmosphere to obtain the final E‐Ni/m‐MgAlO_x_ catalyst. A comparison between the lab‐scale and pilot‐scale syntheses of E‐Ni/m‐MgAlO_x_ catalysts shows that both were designed with consistent theoretical compositions of approximately 5 wt.% Ni, 5 wt.% MgO, and 90 wt.% Al_2_O_3_ (Table S2, Supporting Information). The pilot‐scale synthesis represents an approximately 75‐fold increase in theoretical catalyst mass, from 11.0 to 830.8 g. Despite this substantial scale‐up, the actual product yields remained high, with 92.7% for the lab scale and an impressive 97.5% for the pilot scale, highlighting the excellent scalability and efficiency of the synthesis method.

The HAADF‐STEM, TEM, and elemental mapping analyses of the pilot‐scale E‐Ni/m‐MgAlO_x_ catalyst revealed that Ni nanoparticles were uniformly dispersed within the mesoporous oxide matrix (Figure 7b,c; Figure S12, Supporting Information). Based on measurements of 200 particles, the average diameter of the Ni nanoparticles was determined to be 11.9 nm, slightly larger than that of the lab‐scale catalyst (≈9 nm) (Figure 7d). The XRD pattern of the pilot‐scale catalyst exhibited similar features to the lab‐scale counterpart, with diffraction peaks corresponding to metallic Ni, MgAl_2_O_4_, and NiAl_2_O_4_ phases (Figure 7e). The Ni crystallite size estimated using the Debye‐

Scherrer equation was 11.5 nm, which closely matched the particle size determined by TEM. In large‐scale catalyst synthesis, active particles tend to grow larger due to factors such as non‐uniform heat and mass transfer, less precise mixing, and extended drying or calcination conditions. Nevertheless, this synthesis method achieved effectively controlled particle growth, with the increase in particle size limited to less than 30%, which is considered acceptable for scaled‐up catalytic materials.

**Figure 8:**
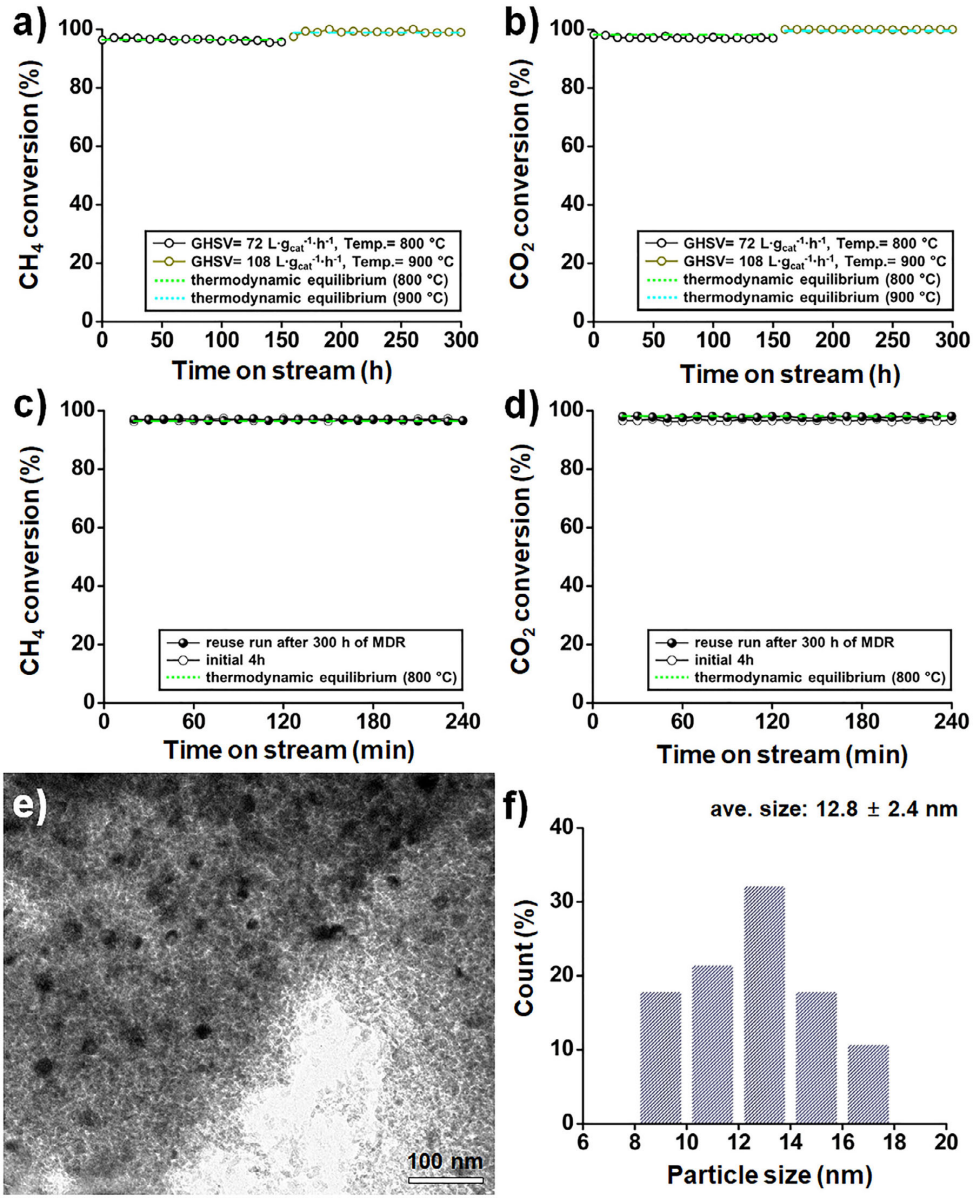
a,b) CH_4_ and CO_2_ conversions for the pilot‐scale E‐Ni/m‐MgAlO_x_ catalyst during long‐term MDR operation (CH_4_/CO_2_/N_2_ = 1:1:8, GHSV = 72 and 108 L·g_cat_^−1^·h^−1^ at 800 and 900 °C, respectively). c,d) CH_4_ and CO_2_ conversions during the reuse test of the pilot‐scale E‐Ni/m‐MgAlO_x_ catalyst under the initial reaction conditions (GHSV = 72 L·g_cat_^−1^·h^−1^) following 300 h of MDR operation. e) Low‐magnification TEM image and f) particle size distribution of Ni nanoparticles in the recovered pilot‐scale E‐Ni/m‐MgAlO_x_ catalyst after 304 h of reaction.

To evaluate the catalytic performance of the pilot‐scale E‐Ni/m‐MgAlO_x_ catalyst, a 300 h continuous MDR reaction was performed. The reaction was initiated at 800 °C with a GHSV of 72 L·g_cat_^−1^·h^−1^ for 150 h, followed immediately by operation at 900 °C and 108 L·g_cat_^−1^·h^−1^ for an additional 150 h without interruption. Throughout the entire 300 h period, both CH_4_ and CO_2_ conversions remained highly stable, maintaining elevated values of approximately 99%, which were close to their respective thermodynamic equilibrium limits (**Figure** **8**a,b). A subsequent reuse test under the initial conditions confirmed negligible deactivation (Figure 8c,d). Post‐reaction TEM analysis showed uniformly dispersed Ni nanoparticles (12.8 ± 2.4 nm) with no significant agglomeration or carbon deposition, indicating excellent resistance to sintering and coking (Figure 8e,f).

When benchmarked against representative Ni‐based MDR catalysts, E‐Ni/m‐MgAlO_x_ exhibits a unique combination of near‐equilibrium conversion, long‐term durability, and proven scalability to the hundred‐gram level (Table S3, Supporting Information). In contrast to dense oxide or alloy exsolution systems with limited surface area and scalability, and other nanostructures confined to lab‐scale performance, this work provides a practical pathway toward the development of industrially viable Ni‐based MDR catalysts.

## 3 Conclusion

In conclusion, we have developed a robust and scalable sol–gel synthesis method for E‐Ni/m‐MgAlO_x_ catalysts, in which exsolved Ni nanoparticles are uniformly embedded within a thermally stable, mesoporous Mg‐modified alumina matrix. This architecture provides strong metal–support interactions, enhanced surface basicity, and excellent resistance to sintering and carbon deposition, enabling outstanding durability under demanding reaction conditions. At the lab scale, the catalyst sustained near‐equilibrium CH_4_ and CO_2_ conversions for 1160 h of continuous MDR operation with negligible deactivation and minimal carbon accumulation. Building on this success, the synthesis was scaled up by ≈75‐fold to the pilot scale, where the catalyst preserved its mesoporous architecture and demonstrated stable MDR performance for 300 h under industrially relevant conditions. These results underscore the practical feasibility of E‐Ni/m‐MgAlO_x_ catalysts for methane dry reforming and establish a scalable exsolution strategy to overcome the long‐standing challenges of Ni‐based systems, offering a promising pathway toward sustainable and industrially viable syngas production.

## Conflict of Interest

The authors declare no conflict of interest.

## Supporting information



Supporting Information

Supplemental Movie 1

Supplemental Movie 2

## Data Availability

The data that support the findings of this study are available from the corresponding author upon reasonable request.
