# Supplementary material for: Scalable Exsolution‐Derived E‐Ni/m‐MgAlOx Catalysts with Anti‐Sintering Stability for Methane Dry Reforming
Source: Small. 2025 Sep 15;21(43):e08028. doi: 10.1002/smll.202508028 (PMC12571206; doi:10.1002/smll.202508028)
Supplement: Supplementary file 1 — Supporting Information [file SMLL-21-e08028-s003.doc]

**Scalable Exsolution-Derived E-Ni/m-MgAlO_x_ Catalysts with Anti-Sintering Stability for Methane Dry Reforming**

Kyung Hee Oh,^[a]^ Shin Wook Kang,^[a]^ Byeong-Seon An,^[b]^ Jung-Il Yang,^[a]^ and Ji Chan Park^[a]^,*

^a^ Clean Fuel Research Laboratory, Korea Institute of Energy Research, Daejeon 34129, Republic of Korea.

^b^ Analysis Center for Energy Research, Korea Institute of Energy Research, Daejeon 34129, Republic of Korea.

*Corresponding author, E-mail: jcpark@kier.re.kr

**Experimental Methods**

**Materials and chemicals**

Nickel(II) nitrate hexahydrate (Ni(NO_3_)_2_·6H_2_O, ≥97%), magnesium nitrate hexahydrate (Mg(NO_3_)_2_·6H_2_O, 99%), aluminum tri-sec-butoxide (Al[OCH(CH_3_)C_2_H_5_]_3_, 97%), and poly(ethylene glycol)-block-poly(propylene glycol)-block-poly(ethylene glycol) (Pluronic P123, PEG-PPG-PEG, Mn ~5,800) were purchased from Aldrich. A 60 wt% aqueous solution of nitric acid (HNO_3_) and anhydrous ethanol (C_2_H_5_OH, ≥99%) were obtained from SAMCHUN. All chemicals were used as received without further purification. For performance comparison, a commercial nickel catalyst (FCR, Süd-Chemie) was used as a reference.

**Synthesis of E-Ni/m-MgAlO_x_**

The E-Ni/m-MgAlO_x_ nanocatalyst was synthesized via a sol–gel method followed by thermal calcination and hydrogen reduction. Initially, 370 mL of distilled water was added to a 1 L polypropylene (PP) bottle and heated in an oil bath at 85 °C for 15 min. Once the temperature stabilized, aluminum tri-sec-butoxide (48.35 g, 196.3 mmol), nickel(II) nitrate hexahydrate (2.48 g, 8.5 mmol), and magnesium nitrate hexahydrate (3.18 g, 12.4 mmol) were sequentially added. The mixture was stirred at 85 °C for 15 min to promote hydrolysis of the aluminum precursor and dissociation of the metal nitrates. To further facilitate hydrolysis, 1 mL of 60 wt% aqueous nitric acid was added dropwise. The mixture was then aged at 85 °C for 1 h with the bottle cap open, followed by another hour of sealed stirring at the same temperature. Subsequently, 36 g of Pluronic P123 was added, and the solution was stirred at 150 rpm for 1 h at 85 °C until a homogeneous sol was obtained. The resulting sol was poured into a glass Petri dish and dried at 80 °C for 24 h to form a gel. The dried gel was transferred to an alumina boat and calcined in ambient air using a tube furnace. The temperature was ramped up to 800 °C over 2 h and held for 8 h, during which the metal precursors were oxidized and Pluronic P123 was thermally decomposed. The decomposition of Pluronic P123 generated mesopores within the oxide matrix, leading to the formation of a mesoporous MgAlO_x_ support. Finally, the calcined powder was reduced under a continuous flow of hydrogen gas. The temperature was raised to 800 °C over 2 h and held for 4 h, inducing exsolution of metallic nickel from the oxide matrix. To prevent surface oxidation upon air exposure, the reduced E-Ni/m-MgAlO_x_ catalyst was passivated by immersion in anhydrous ethanol prior to storage.

**Synthesis of low-Ni-loading (1 wt%) E-Ni/m-MgAlO_x_ catalyst**

To investigate the effect of low Ni loading on catalyst performance, an E-Ni/m-MgAlO_x_ catalyst containing 1 wt% Ni was synthesized using the same sol-gel method described above. Nickel(II) nitrate hexahydrate (0.53 g, 1.8 mmol), magnesium nitrate hexahydrate (3.39 g, 13.2 mmol), and aluminum tri-sec-butoxide (48.35 g, 196.3 mmol) were sequentially added to 370 mL of distilled water preheated to 85 °C in a 1 L PP bottle. The mixture was stirred at 85 °C for 15 min to initiate hydrolysis and precursor dissolution. Next, 1 mL of 60 wt% nitric acid was added dropwise to further promote hydrolysis, followed by aging at 85 °C for 1 h with the bottle open. Subsequently, 36 g of Pluronic P123 was added, and the solution was stirred at 150 rpm for 1 h at 85 °C until a homogeneous sol was formed. The sol was poured into a glass Petri dish and dried at 80 °C for 24 h to form a gel. The dried gel was calcined in ambient air by ramping the temperature to 800 °C over 2 h, followed by an 8 h hold at the same temperature to produce mesostructured MgAlO_x_. The calcined powder was then reduced under a hydrogen flow at 800 °C for 4 h to induce exsolution of Ni nanoparticles. To prevent surface oxidation, the reduced catalyst was passivated in anhydrous ethanol immediately after thermal treatment.

**Synthesis of E-Ni/m-Al_2_O_3_**

The E-Ni/m-Al_2_O_3_ nanocatalyst was synthesized via a sol-gel method, followed by thermal calcination and hydrogen reduction. Aluminum tri-sec-butoxide (48.35 g, 196.3 mmol) and nickel(II) nitrate hexahydrate (2.35 g, 8.1 mmol) were sequentially added to 370 mL of distilled water preheated to 85 °C in a 1 L PP bottle. The mixture was stirred at 85 °C for 15 min to initiate hydrolysis and dissolution of the metal precursors. Subsequently, 1 mL of 60 wt% aqueous nitric acid was added dropwise to further promote hydrolysis. The mixture was aged at 85 °C for 3 h with the bottle cap open, followed by an additional 21 h with the bottle sealed. Pluronic P123 (36 g) was then added, and the solution was stirred at 150 rpm for 24 h at 85 °C to obtain a homogeneous sol. The resulting sol was poured into a glass Petri dish and dried at 80 °C for 24 h to induce gelation. The dried gel was calcined in ambient air by ramping the temperature to 800 °C over 2 h and holding it for 8 h. During this process, the Pluronic P123 template was decomposed, yielding a mesostructured NiAlO_x_ support. Finally, the calcined powder was reduced under a continuous flow of hydrogen gas by heating to 800 °C over 2 h, followed by a 4 h hold at that temperature, resulting in the exsolution of metallic Ni from the oxide matrix. To prevent surface oxidation upon air exposure, the reduced E-Ni/m-Al_2_O_3_ catalyst was passivated by immersion in anhydrous ethanol before storage.

**Pilot-scale synthesis of E-Ni/m-MgAlO_x_**

To validate the scalability of the E-Ni/m-MgAlO_x_ nanocatalyst, the synthesis was scaled up by a factor of 75 using a 60 L hydrothermal reactor, following the same sol-gel-based protocol employed at the laboratory scale. Aluminum tri-sec-butoxide (3,626 g, 14.7 mol), nickel(II) nitrate hexahydrate (198.9 g, 0.684 mol), and magnesium nitrate hexahydrate (255.4 g, 0.996 mol) were sequentially added to 15 L of distilled water preheated to 70 °C. The mixture was stirred at 70 °C for 15 min to initiate hydrolysis and salt dissociation. To further promote hydrolysis, a 60 wt% aqueous nitric acid solution was added dropwise, followed by continuous stirring at 70 °C for 1 h. Pluronic P123 (2,700 g), pre-dissolved in 15 L of distilled water, was then added, and the resulting solution was stirred at 300 rpm for 2 h to stabilize the sol. The homogeneous sol was poured into stainless-steel trays and dried at 80 °C for 24 h to form a gel. The dried gel was calcined in a tube furnace under ambient air by ramping the temperature to 800 °C over 2 h, followed by an 8 h isothermal hold. During this step, the Pluronic P123 template was thermally decomposed, yielding a mesostructured m-NiMgAlO_x_ oxide matrix. To ensure uniform thermal treatment throughout the bulk sample, a secondary calcination was performed in a rotary furnace operating at 10 rpm. The resulting powder was subsequently reduced under a continuous flow of hydrogen gas at 800 °C for 4 h. To prevent surface oxidation during recovery and storage, the reduced catalyst was passivated by immersion in anhydrous ethanol.

***In situ* TEM measurements for thermal stability evaluation**

The thermal stability of the E-Ni/m-Al_2_O_3_ catalyst was evaluated using *in situ* transmission electron microscopy (TEM) under controlled heating conditions. A micro-electro-mechanical systems (MEMS)-based heating chip was employed to support the catalyst sample, enabling real-time observation during thermal treatment. The temperature was linearly ramped from 30 °C to 900 °C over 870 s. Once the target temperature was reached, the sample was held at 900 °C for 2,655 s under a 4% H₂/Ar gas atmosphere inside the in-situ TEM chamber. This setup allowed for direct visualization of the catalyst's morphological and structural evolution during heating, providing detailed insights into its thermal robustness and sintering resistance.

**MDR catalytic performance evaluation**

The catalytic performance was evaluated using a custom-designed fixed-bed reactor system equipped with a quartz reactor tube (1/4 inch in diameter), real-time monitoring capabilities, and a programmable automation controller to ensure safe, long-term operation. Prior to testing, in-situ reduction was carried out by ramping the catalyst temperature to 800 °C over 20 min under a hydrogen flow of 100 mL·min^-1^ at atmospheric pressure, followed by an isothermal hold at 800 °C for 40 min. For the methane dry reforming (MDR) reaction, the feed gases (CH_4_, CO_2_, and N_2_) were precisely regulated using mass flow controllers (MFCs) and preheated via a band-type line heater maintained at 200 °C before entering the reactor. Reactions were carried out using 50 mg of catalyst under various conditions, including a CH_4_/CO_2_ feed ratio of 1, with or without N₂ dilution, and gas hourly space velocities (GHSV) ranging from 54 to 144 L·g_cat_^-1^·h^-1^. To ensure safety during extended high-temperature operation, an automatic shutdown system was implemented to continuously monitor key parameters such as gas flow rate and reactor pressure. Fail-safe protocols, including overpressure shutoff, low-flow shutdown, and communication failure detection, were integrated into and managed by the central controller. Catalyst stability was assessed over 1,164 hours of continuous time-on-stream (TOS) operation under progressively harsher GHSV conditions. Product gases were analyzed using an online gas chromatography system (iGC7200, DS Science Inc.) equipped with a thermal conductivity detector (TCD), and total flow was measured using a calibrated wet gas meter (Shinagawa Co.).

**Characterization**

Transmission electron microscopy (TEM) images were acquired using a Talos F200X microscope operated at 200 kV. Elemental mapping was conducted via energy-dispersive X-ray spectroscopy (EDS) using a Super-X detection system equipped with four windowless silicon drift detectors (SDDs) for high-efficiency detection. In-situ TEM analysis was conducted using a Hitachi HF5000 microscope, also operated at 200 kV. X-ray diffraction (XRD) patterns were recorded on a Rigaku SmartLab high-power powder diffractometer (9 kW) over a 2*θ* range of 20–80°. Nitrogen sorption isotherms were measured at –196 °C using a TriStar II 3020 surface area analyzer. Prior to measurement, samples were degassed under vacuum at 300 °C for 4 h. X-ray photoelectron spectroscopy (XPS) was performed using a Thermo Scientific Nexsa G2 system equipped with a monochromatic Al Kα X-ray source. Nickel and magnesium contents were quantified by inductively coupled plasma optical emission spectrometry (ICP-OES) using a PerkinElmer AVIO 500 system. Temperature-programmed desorption (TPD) of ammonia (NH_3_) and carbon dioxide (CO_2_) was carried out using a Micromeritics Autochem II 2920 instrument. Prior to the NH_3_-TPD experiment, the samples were pretreated at 400 °C for 1 h under a 50 mL·min^-1^ flow of pure H_2_ to remove adsorbed water, followed by saturation with 15% NH₃ in He (50 mL·min^-1^) at 100 °C for 1 h. NH_3_ desorption was then carried out from 100 to 700 °C at a heating rate of 10 °C·min^-1^ under a pure He flow. For CO_2_-TPD, the samples were pretreated in He (50 mL·min^-1^) at 500 °C for 1 h to remove adsorbed species, cooled to 50 °C, and exposed to a 10% CO_2_/He mixture for 2 h. After purging with pure He, desorption was conducted from 50 to 700 °C at 10 °C·min^-1^ under a He flow of 50 mL·min^-1^. Carbon deposition was quantified by thermogravimetric analysis (TGA, Setaram) using a heating rate of 10 °C·min^-1^ from 30 to 800 °C under an air flow of 10 mL·min⁻¹. Raman spectroscopy was performed on a Horiba LabRAM HR Evolution microscope with a 514 nm excitation laser


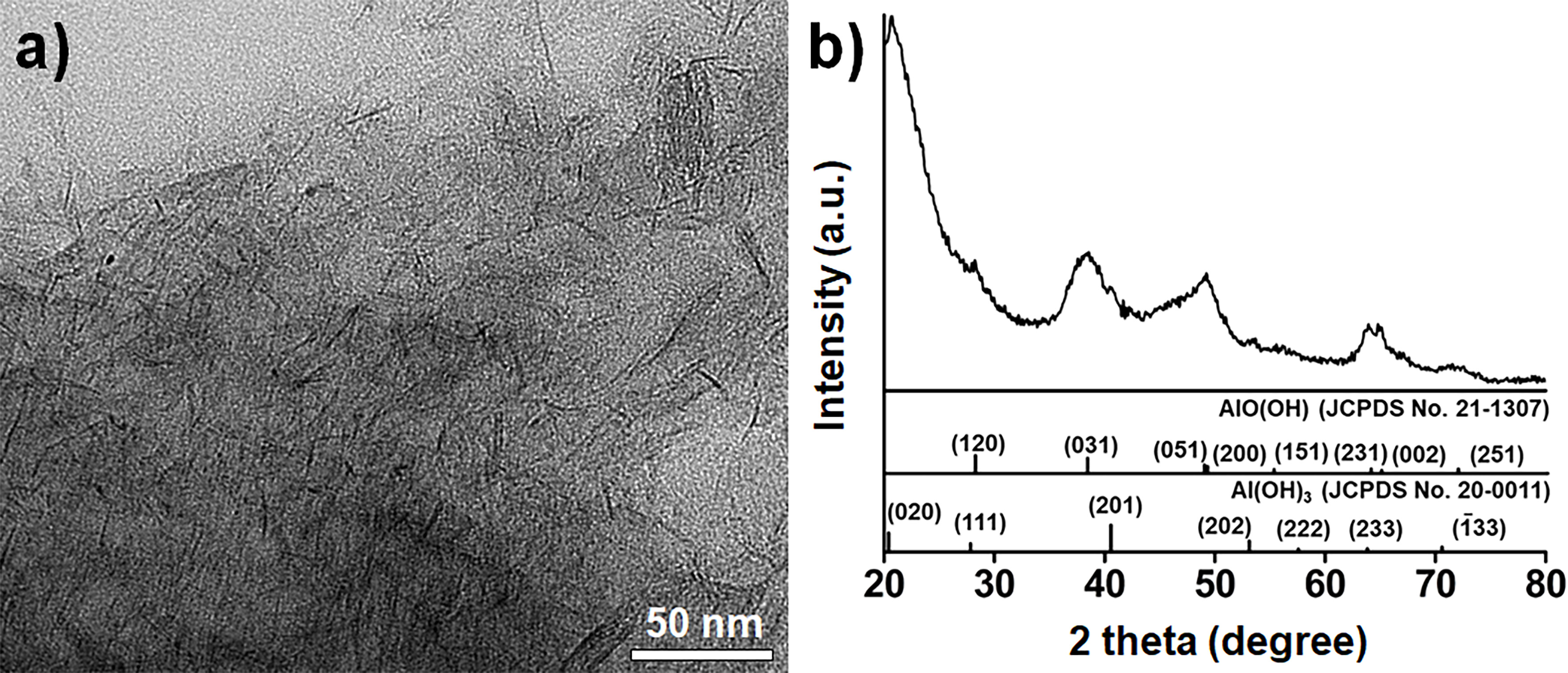


**Figure S1.** (a) TEM image and (b) XRD pattern of the Ni–Mg–Al hydroxide (NMA) gel.


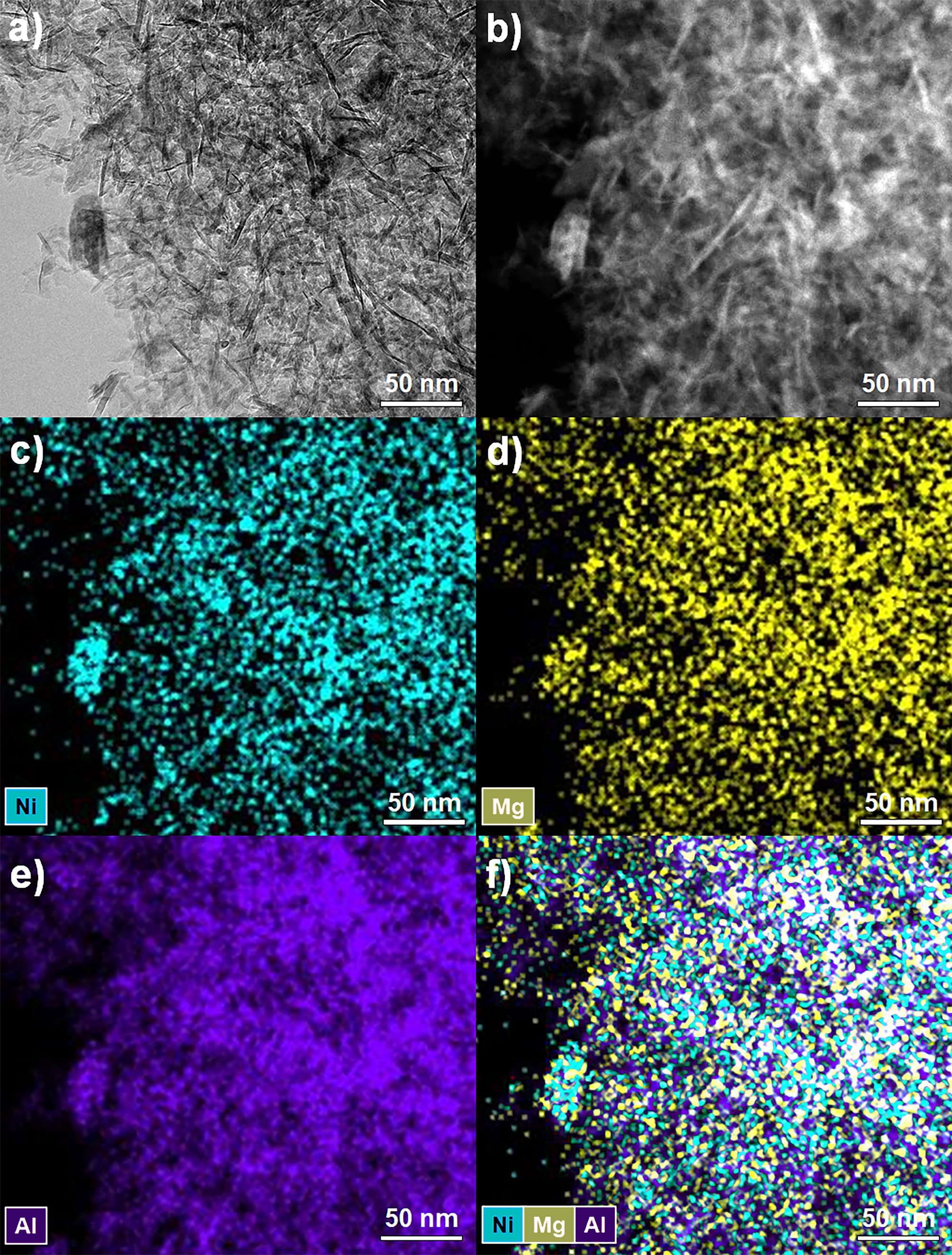


**Figure S2.** (a) TEM image and (b) high-angle annular dark-field scanning TEM (HAADF-STEM) image of m-NiMgAlOx, with corresponding elemental mapping images of (c-e) Ni, Mg, and Al, and (f) the overlay map.


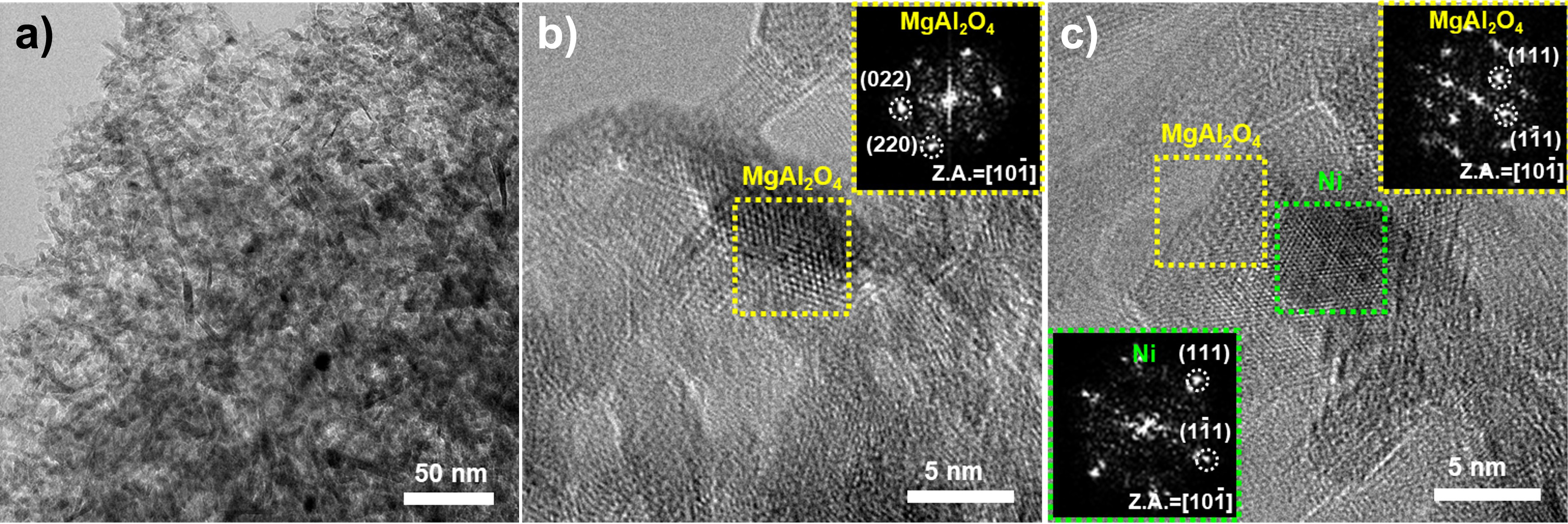


**Figure S3**. (a) TEM image and (b, c) HR-TEM images of the E-Ni/m-MgAlOₓ catalyst, with corresponding FFT patterns from the highlighted regions.


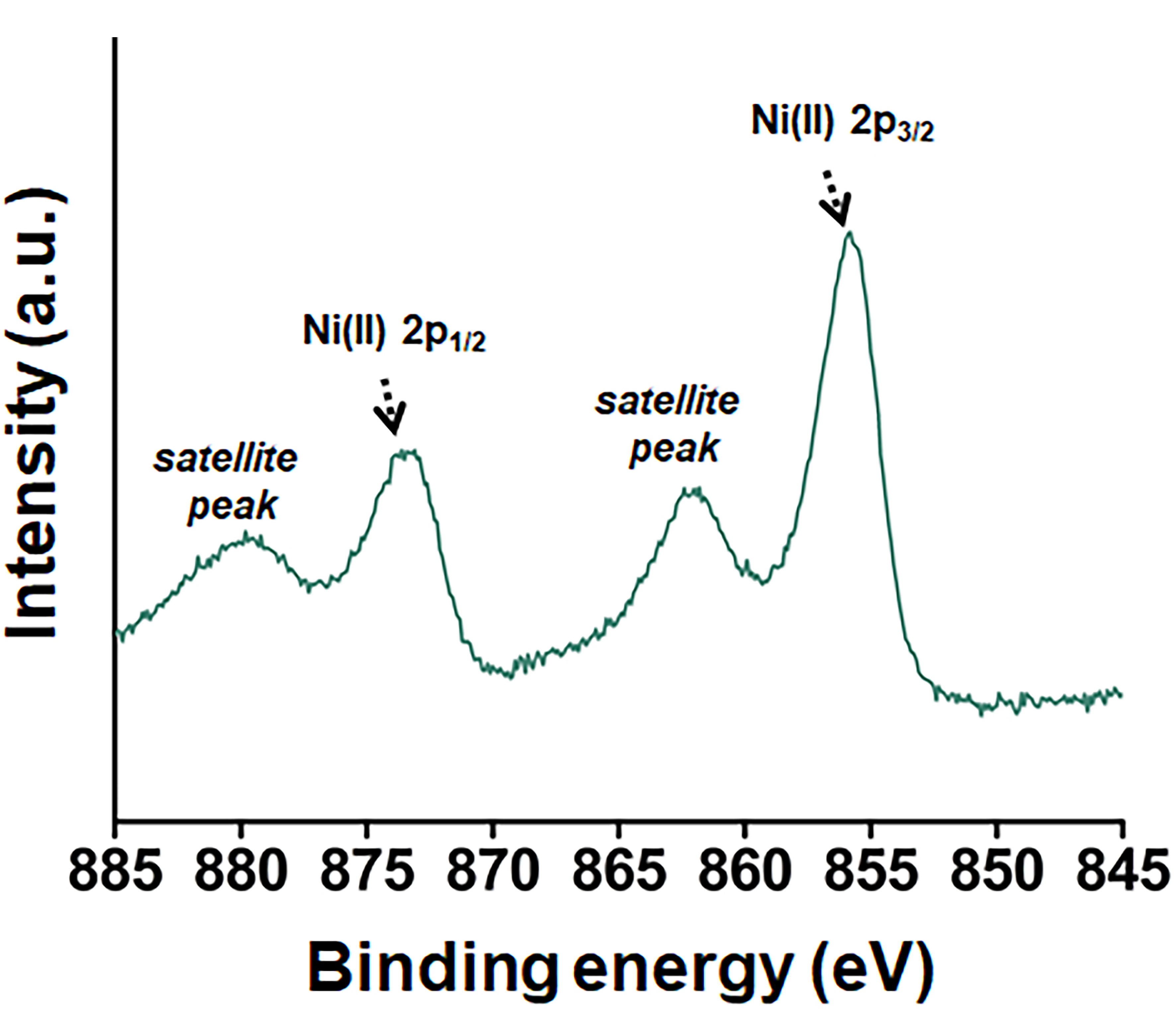


**Figure S4**. XPS spectrum of m-NiMgAlO_x_ in the Ni 2p binding energy region.


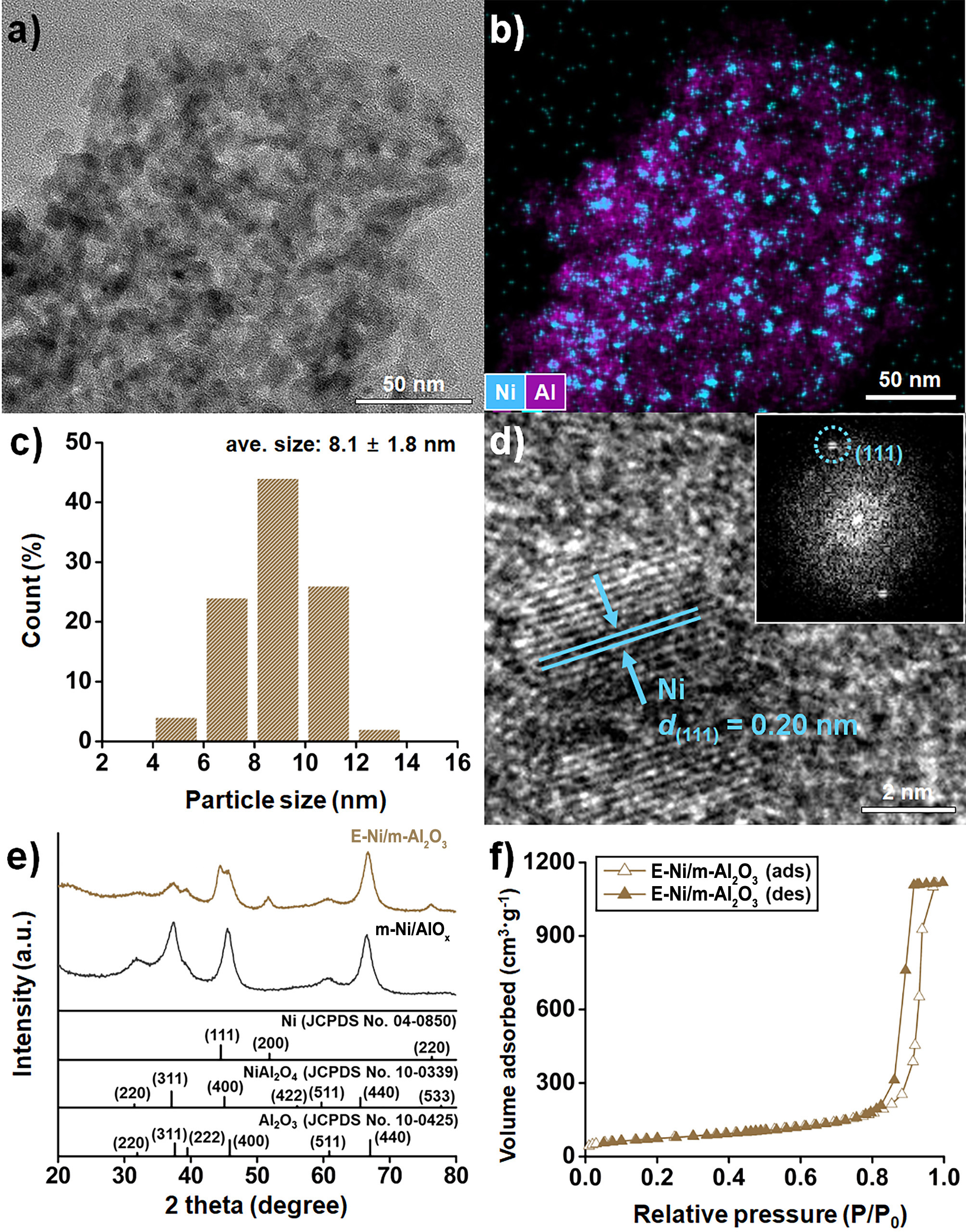


**Figure S5.** (a) TEM image, (b) elemental mapping images of Ni and Al, (c) Ni particle size distribution diagram, and (d) HR-TEM image with the corresponding FFT electron diffraction pattern (inset) of E-Ni/m-Al_2_O_3_. (e) XRD patterns of E-Ni/m-Al_2_O_3_ and m-NiAlO_x_, and (f) N_2_ sorption isotherms of E-Ni/m-Al_2_O_3_.


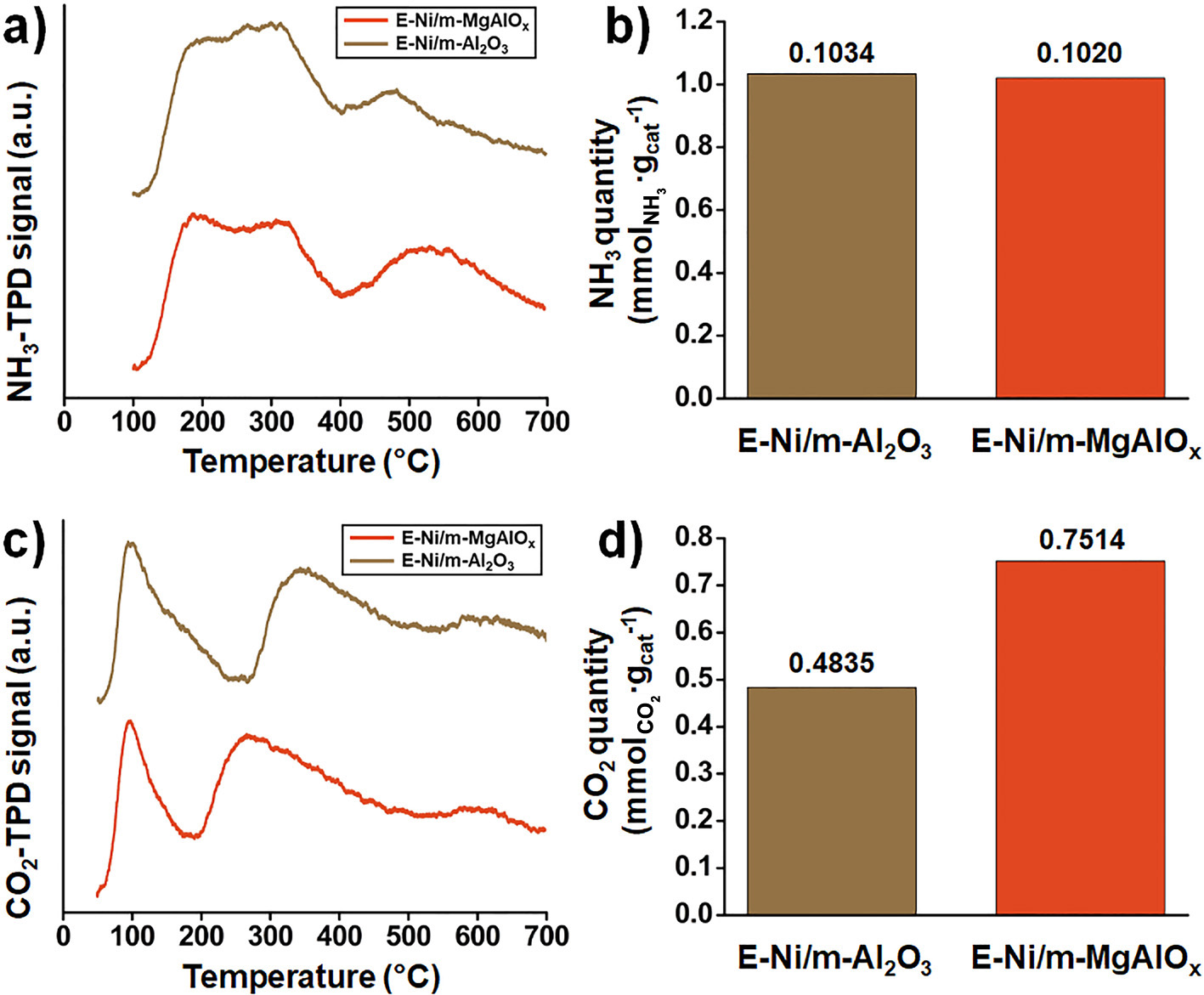


**Figure S6.** (a) NH_3_-TPD profiles of E-Ni/m-Al_2_O_3_ and E-Ni/m-MgAlO_x_ catalysts, (b) corresponding NH_3_ uptake quantities, (c) CO_2_-TPD profiles of E-Ni/m-Al_2_O_3_ and E-Ni/m-MgAlO_x_ catalysts, and (d) corresponding CO_2_ uptake quantities.


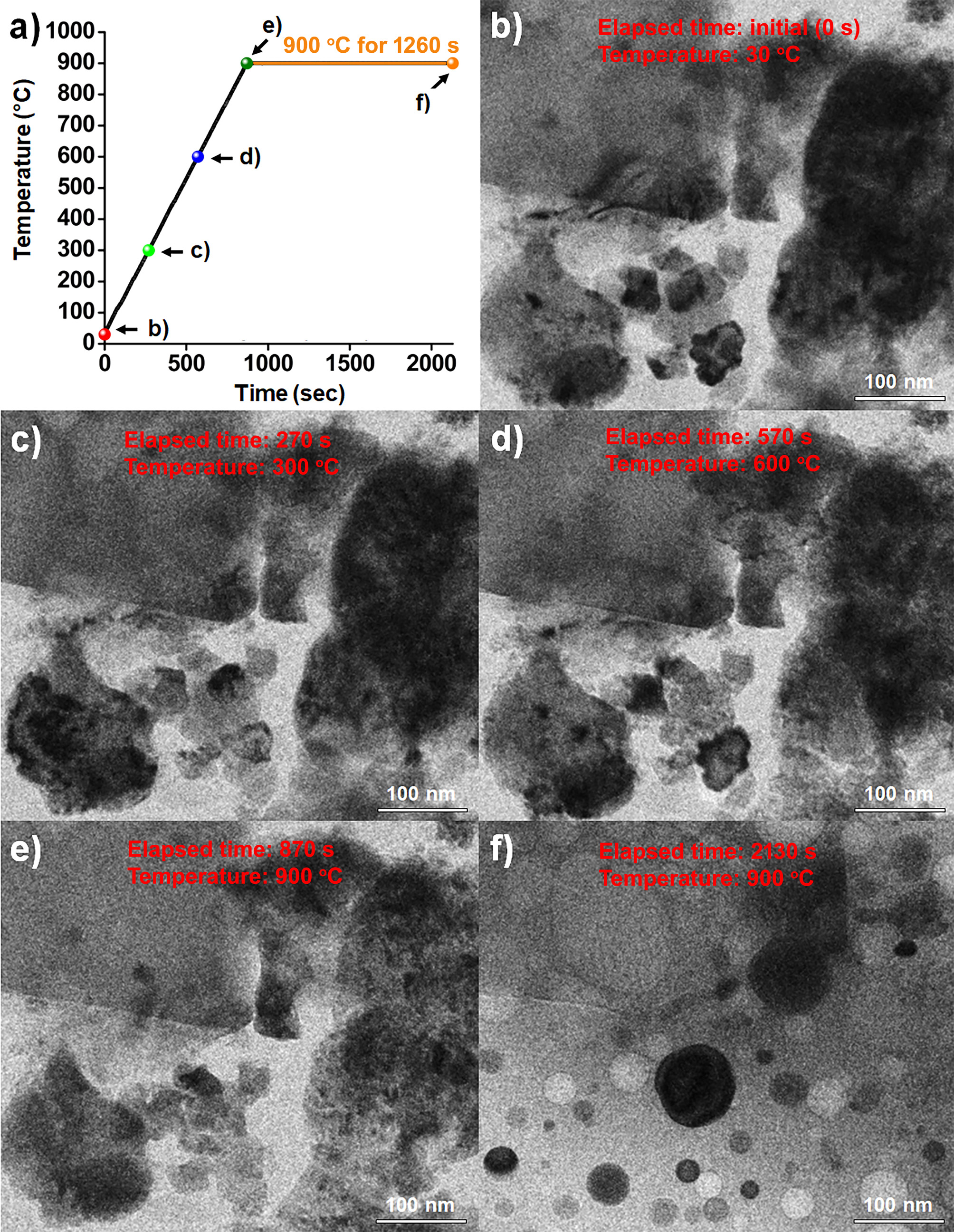


**Figure S7**. (a) Temperature profile during the *in situ* TEM heating experiment. (b-f) *In situ* TEM images of the commercial Ni catalyst acquired at different temperatures and elapsed times.


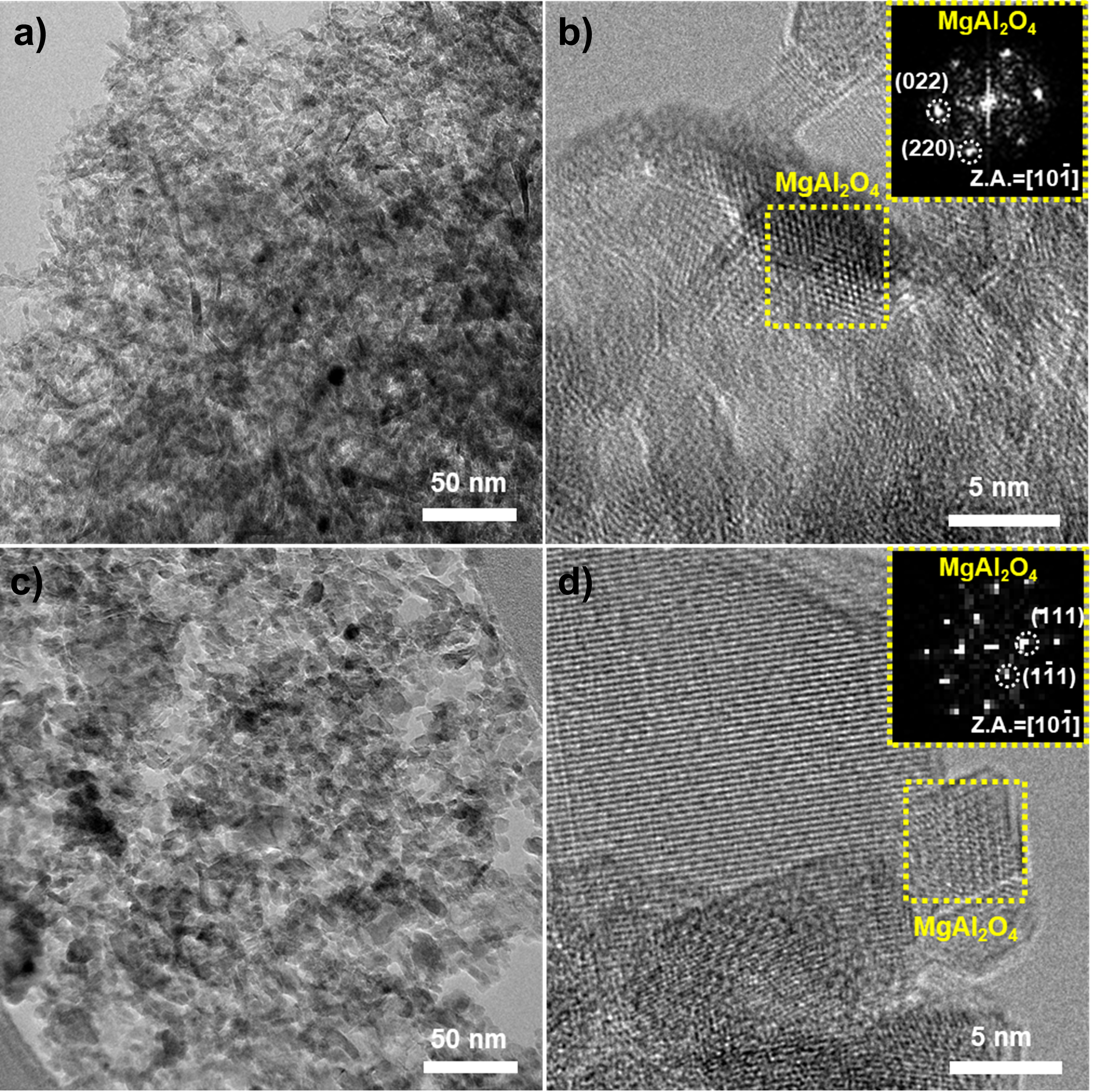


**Figure S8**. TEM and HR-TEM images of (a, b) the fresh E-Ni/m-MgAlO_x_ catalyst with corresponding FFTs and (c, d) the catalyst recovered after 1,164 h of reaction.


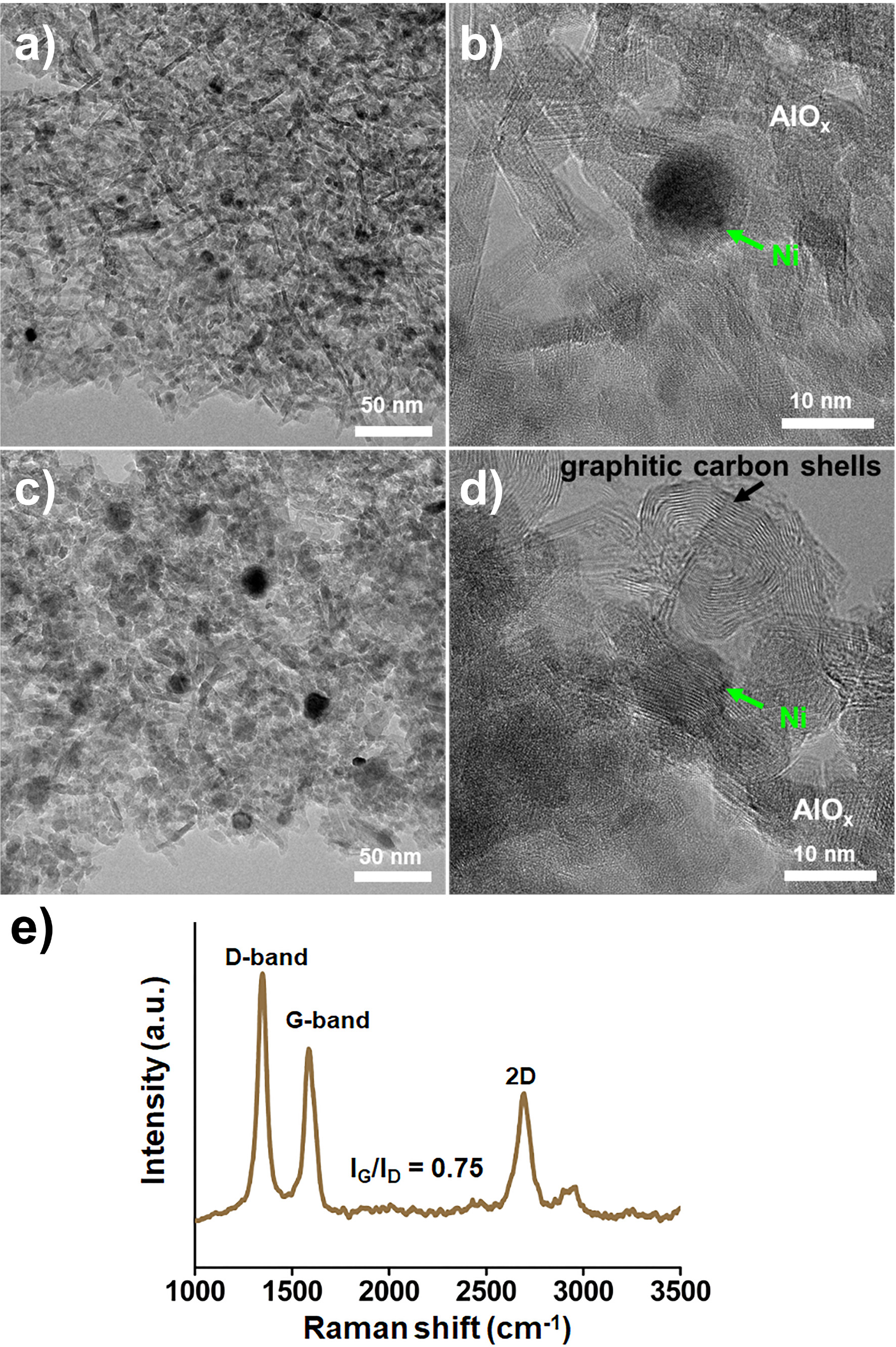


**Figure S9.** (a) TEM and (b) HR-TEM images of the fresh E-Ni/m-Al_2_O_3_ catalyst, and (c) TEM, (d) HR-TEM images, and (e) Raman spectrum of the E-Ni/m-Al_2_O_3_ catalyst after 500 h of reaction.


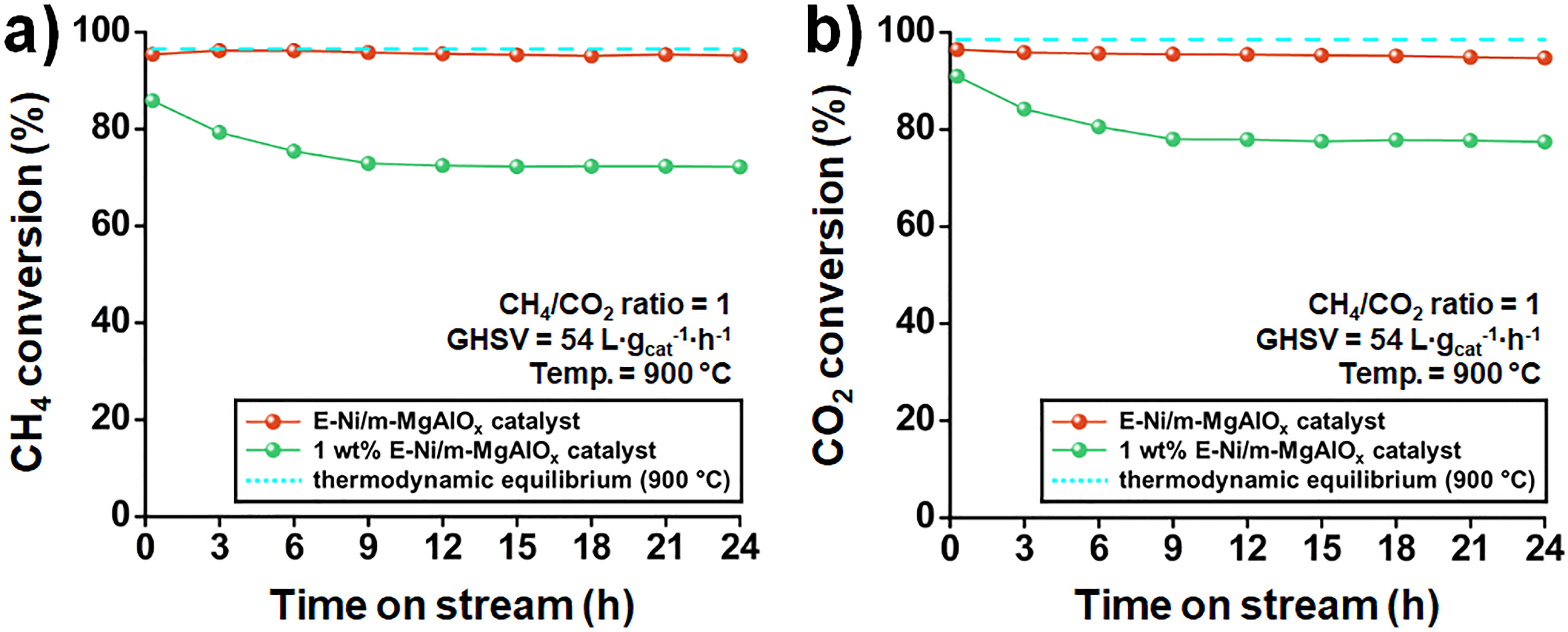


**Figure S10.** Catalytic performance of a low-Ni-loading E-Ni/m-MgAlO_x_ catalyst (ca. 1 wt% Ni) compared with the standard E-Ni/m-MgAlO_x_ catalyst (ca. 5 wt% Ni) under undiluted MDR conditions (CH_4_/CO_2_ = 1:1, GHSV = 54 L·g_cat_^-1^·h^-1^, 900 °C): (a) CH_4_ conversion and (b) CO_2_ conversion.


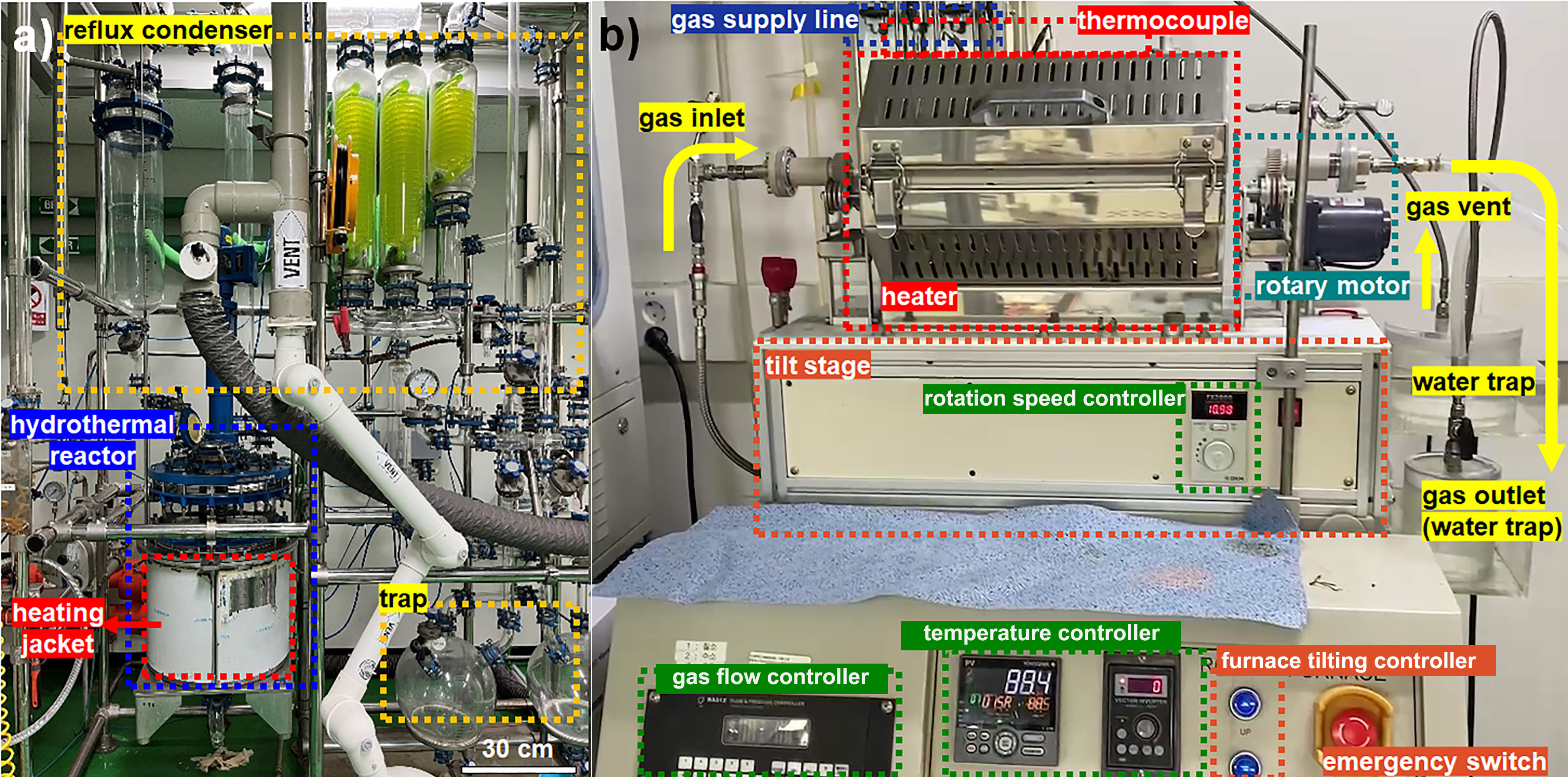


**Figure S11.** Photographs of (a) the pilot-scale hydrothermal reactor and (b) the large-scale rotary furnace used for thermal treatment in the pilot-scale synthesis.


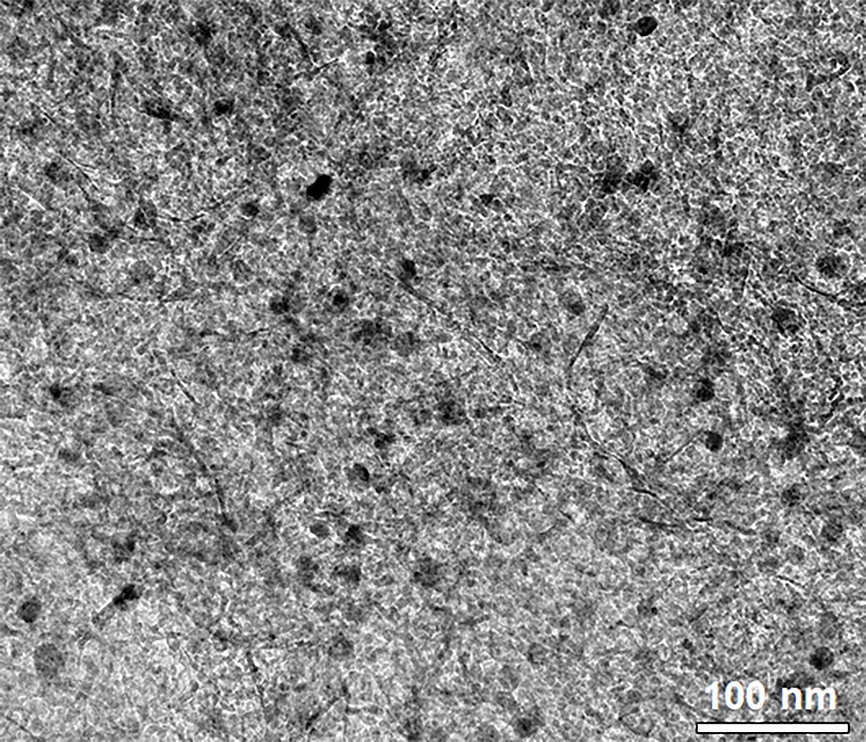


**Figure S12.** TEM image of the pilot-scale E-Ni/m-MgAlO_x_ catalyst.

**Table S1**. Comparison of greenhouse gas conversion capacities per unit mass of catalyst for representative MDR catalysts reported in the literature, based on long-term reaction results (>200 h) under various reaction conditions.

| **catalyst** | **GHSV**  **(L∙g_cat_^-1^∙h^-1^)** | **CH_4_/CO_2_/**  **inert gas** | **rxn. temp. (℃)** | **rxn. time (h)** | **final CH_4_ conv. (%)** | **final CO_2_ conv. (%)** | **total amount of reactants converted**  **(L·g_cat_^-1^)** | **Ref.** |
| --- | --- | --- | --- | --- | --- | --- | --- | --- |
| **E-Ni/m-MgAlO_x_ (overall reaction)** | **72-144** | **1/1/8** | **800-900** | **1,164** | **95-99** | **95-99** | **20,316** | This work |
| E-Ni/m-MgAlO_x_ (detailed reactions) | 72 | 1/1/8 | 800 | 500 | 96 | 96 | 6,939 |  |
|  | 84 |  | 850 | 300 | 95 | 95 | 4,808 |  |
|  | 96 |  | 900 | 60 | 99 | 99 | 1,140 |  |
|  | 108 |  | 900 | 150 | 98 | 99 | 3,197 |  |
|  | 144 |  | 900 | 150 | 96 | 98 | 4,176 |  |
|  | 72 |  | 800 | 4 | 95 | 97 | 56 |  |
| E-Ni/m-Al_2_O_3_ | 72 | 1/1/8 | 800 | 500 | 84 | 88 | 6,750 |  |
| commercial Ni catalyst | 72 | 1/1/8 | 800 | 200 | 24 | 42 | 1,698 |  |
| NiMo-MgO (NiMoCat) | 60 | 1/1/8 | 800 | 850 | 98 | 100 | 10,098 | [1] |
|  | 300 |  |  | 400 | 72 | 81 | 18,360 |  |
| LCFN55 | 30 | 1/1 | 850 | 1,000 | 92 | 96 | ca. 28,125^a^ | [2] |
| E-Ni0.15Fe0.05 | 30 | 1/1.03/1 | 800 | 260 | 52 | 58 | ca. 2,877^a^ | [3] |
| Ni/SBA-15  /Al_2_O_3_/FeCrAl | 20 | 1/1 | 800 | 1,400 | 89 | 89 | ca. 24,864^a^ | [4] |
| NiEs  (Ni embedded in SiO_2_) | 250 | 3/3/4 | 800 | 500 | 96 | 97 | ca. 72,375^a^ | [5] |
| Ni-MgAl_2_O_4_ | 120 | 1/1/4.66 | 750 | 450 | 90 | 90 | ca. 14,580^a^ | [6] |
| 5Ni-15Co/SBA-15 | 60 | 1/1/3 | 700 | 200 | 75 | 79 | ca. 3,684^a^ | [7] |

^a^Calculated based on literature data. Total amount of reactants converted (L∙g_cat_^-1^) was estimated using inlet flow rates of CH_4_ and CO_2_ (F_CH_4, in_ and F_CO_2, in_), reaction time (t), and conversions X_CH_4_ and X_CO_2_, where X = (F_in_ – F_out_)/F_in_ × 100.

# References)

[1] Y. Song, E. Ozdemir, S. Ramesh, A. Adishev, S. Subramanian, A. Harale, M. Albuali, B. A. Fadhel, A. Jamal, D. Moon, S. H. Choi, C. T. Yavuz, Dry reforming of methane by stable Ni–Mo nanocatalysts on single-crystalline MgO. *Science* **2020**, *367*, 777–781.

[2] J. Oh, S. Joo, C. Lim, H. J. Kim, F. Ciucci, J. Q. Wang, J. W. Han, G. Kim, Precise modulation of triple‐phase boundaries towards a highly functional exsolved catalyst for dry reforming of methane under a dilution‐free system. *Angew. Chem. Int. Ed.* **2022**, *61*, e202204990.

[3] X. Yao, Q. Cheng, Y. Attada, S. Ould-Chikh, A. Ramírez, X. Bai, H. O. Mohamed, G. Li, G. Shterk, L. Zheng, J. Gascon, Y. Han, O. M. Bakr, P. Castaño, Atypical stability of exsolved Ni-Fe alloy nanoparticles on double layered perovskite for CO_2_ dry reforming of methane. *Appl. Catal. B* **2023**, *328*, 122479.

[4] K. Wang, X. Li, S. Ji, B. Huang, C. Li, Preparation of Ni‐Based Metal Monolithic Catalysts and a Study of Their Performance in Methane Reforming with CO2. *ChemSusChem* **2008**, *1*, 527–533.

[5] H. Min, Y. Ju, Y. J. Ji, D. Y. Kim, Y. J. Kim, S. B. Kang, Long-term stable catalyst for dry reforming of Methane: Ni-Nanocluster embedded in silica. *Chem. Eng. J.* **2024**, *489*, 151520.

[6] F. Wen, C. Xu, N. Huang, T. Wang, X. Sun, H. Li, R. Zhang, G. Xia, Exceptional stability of spinel Ni–MgAl2O4 catalyst with ordered mesoporous structure for dry reforming of methane. *Int. J. Hydrogen Energy* **2024**, *69*, 1481–1491.

[7] M. Chaghouri, C. Ciotonea, M. Mohamad Ali, M. Marinova, P. Simon, E. Abi-Aad, S. Royer, C. Gennequin, Deposition precipitation derived Ni-Co active sites for enhanced dry reforming of methane performances. *Catal. Today* **2024**, *429*, 114458.

**Table S2.** Theoretical compositions and actual yields of E-Ni/m-MgAlO_x_ catalysts synthesized at laboratory and pilot scales.

| **catalyst** | **theoretical amount (g)** | | | | **theoretical portion (wt%)** | | | | **actual catalyst**  **mass (g)** | **actual product yield (%)** |
| --- | --- | --- | --- | --- | --- | --- | --- | --- | --- | --- |
|  | Ni | MgO | Al_2_O_3_ | Total | Ni | MgO | Al_2_O_3_ | Total |  |  |
| lab-scale  E-Ni/m-MgAlOₓ | 0.50 | 0.50 | 10.0 | 11.0 | 4.54 | 4.54 | 90.92 | 100.00 | 10.2 | 92.7 |
| pilot-scale  E-Ni/m-MgAlOₓ | 40.15 | 40.14 | 750.51 | 830.8 | 4.83 | 4.83 | 90.34 | 100.00 | 810.0 | 97.5 |

*Theoretical amounts (g) were calculated based on the stoichiometric yields of metals and metal oxides expected from the thermal decomposition of the corresponding metal precursors.

**Table S3**. Representative Ni-based catalyst systems for MDR.

| **catalyst system** | **performance & stability** | **synthesis method** | **scalability / remarks** | **Ref.** |
| --- | --- | --- | --- | --- |
| E-Ni/m-MgAlO_x_ | >95% conv., >1,000 h stability (near-eq.) | sol–gel exsolution in mesoporous alumina | scaled up to hundreds of grams (~75×), high surface area and good diffusion | This work |
| Ni_x_Al_1_O_2-δ_ mesoporous catalysts | high long‑term stability & anti‑coking | exsolution from spinel | dense structure, limited surface area | [1] |
| Ni-Co alloy catalyst | enhanced anti-coking via alloy exsolution | exsolution of Ni–Co alloy | alloy tuning, scalability not demonstrated | [2] |
| Ni-nanocluster in SiO_2_ matrix | stable over 500 h without deactivation | embedding in bimodal silica | lab-scale, mass transport limitations likely | [3] |
| La_0.8_Ce_0.1_Ti_0.6_Ni_0.4_O_3_ (LCTN) | ~85% CH_4_ conversion, 60% CO conversion, controlled Ni size 10–49 nm | Ce-doped perovskite exsolution | lab-scale, scalability not demonstrated | [4] |
| Hollow Ni-ZrO_2_ nanoshell catalysts | highly stable and efficient MDR catalysts | flame aerosol + exsolution method | novel nanoshell, scalability under investigation | [5] |
| NiGa/ZrO_2_ | improved stability (250 h) and enhanced coke resistance | sol-gel exsolution | lab-scale, limited by dispersion and scalability not demonstrated | [6] |
| Pr-promoted Ni-Mg-Al(O) | high thermal stability, 45-51% of CH_4_ conversion 35-38% of CO_2_ conversion | microwave -assisted self-combustion + exsolution method | lab-scale, lacks clear advantages over existing CeO_2_-based systems | [7] |
| Ni single atom on mesoporous CeO_2_ | 91% CH_4_ conversion and 90% CO_2_ conversion for 120 h | SBA-15 silica  as hard template + impregnation method | scalability not demonstrated, lack of thermal stability at high temperatures | [8] |

# References)

[1] S. Zhang, M. Ying, J. Yu, W. Zhan, L. Wang, Y. Guo, Y. Guo, Ni_x_Al_1_O_2-δ_ mesoporous catalysts for dry reforming of methane: The special role of NiAl_2_O_4_ spinel phase and its reaction mechanism. *Appl. Catal. B* **2021**, *291*, 120074.

[2] D. H. Kim, J.-C. Seo, Y. J. Kim, J. Kim, S. Yoon, H. Ra, M.-J. Kim, K. Lee, Ni-Co alloy catalyst derived from NixCo_y_/MgAl_2_O_4_ via exsolution method for high coke resistance toward dry reforming of methane. *Catal. Today* **2024**, *425*, 114337.

[3] H. Min, Y. Ju, Y. J. Ji, D. Y. Kim, Y. J. Kim, S. B. Kang, Long-term stable catalyst for dry reforming of Methane: Ni-Nanocluster embedded in silica. *Chem. Eng. J.* **2024**, *489*, 151520.

[4] H. Kim, R. Mane, K. Han, H. Kim, C. Lee, Y. Jeon, In Situ Control of the Eluted Ni Nanoparticles from Highly Doped Perovskite for Effective Methane Dry Reforming. *Nanomaterials* **2022**, *12*, 3325.

[5] S. Liu, C. Dun, M. Shah, J. Chen, S. Rao, J. Wei, E. A. Kyriakidou, J. J. Urban, M. T. Swihart, Producing ultrastable Ni-ZrO_2_ nanoshell catalysts for dry reforming of methane by flame synthesis and Ni exsolution. *Chem Catalysis* **2022**, *2*, 2262–2274.

[6] Z. Yu, Z. Liu, Z. Chen, Z. Wang, X. Zheng, S. Yang, L. Tan, H. Xu, L. Wang, H. Guo, Q. Wei, Promotion of Ga in Ni/ZrO_2_ catalyst for ultra-stable and coke-resisting dry reforming of methane. *Fuel* **2025**, *399*, 135690.

[7] O. Ojeda-Niño, J. Gallego, C.E. Daza, Pr-Promoted Ni Exsolution from Ni–Mg–Al (O) as Catalysts for Syngas Production by Dry Reforming of Methane. *Results in Engineering* **2023**, *17*, 100821.

[8] J. Li, C. Du, Q. Feng, Y. Zhao, S. Liu, J. Xu, M. Hu, Z. Zeng, Z. Zhang, H. Shen, Y. Zhang, J. Zhu, L. Zhang, W. Zhao, J. Huang, H. Xiong, Evolution and performances of Ni single atoms trapped by mesoporous ceria in Dry Reforming of Methane. *Appl. Catal. B* **2024**, *354*, 124069.

**Captions for Supplementary Videos**

**Movie S1.** *In situ* transmission electron microscopy (TEM) analysis of the E-Ni/m-MgAlO_x_ catalyst during heating from 30 °C to 900 °C followed by an isothermal hold, demonstrating its thermal stability.

**Movie S2.** *In situ* TEM analysis of the commercial Ni catalyst under identical conditions for comparison.
